# Handcrafted Features Versus Learned Representations for IMU-Based Goat Behavior Classification: Accuracy, Validation Design, and Computational Cost

**DOI:** 10.3390/s26206424

**Published:** 2026-10-11

**Authors:** Xue-Cheng Jin, Seong-Jin Kim, Ji-Hoon Lee, Yoon-Chul Choi, Ju-Hyeon Kim, Rajaraman Bharanidharan, Na-Yeon Kim

**Affiliations:** 1 Asia Pacific Ruminant Institute, Icheon 17385, Republic of Korea; kimhs@apri.kr (X.-C.J.); apri76@apri.kr (S.-J.K.); 2 Bodit Co., Ltd., Seoul 08594, Republic of Korea; mark.lee@bodit.co (J.-H.L.); kevin.choi@bodit.co (Y.-C.C.); bley@bodit.co (J.-H.K.); 3 Department of Eco-Friendly Livestock Science, Institutes of Green Bio Science and Technology, Seoul National University, Pyeongchang 25354, Republic of Korea; bharanidharan7@snu.ac.kr

**Keywords:** goat, rumination, eating behavior, accelerometer, representation learning, leave-one-subject-out, inference latency, precision livestock farming

## Abstract

**Highlights:**

**What are the main findings?**

- A 24-dimensional learned representation matched a 250-dimensional handcrafted feature set, and two structurally unrelated feature extractors reached the same accuracy. What mattered most was the training objective, not the architecture.
- Window-level cross-validation overestimated accuracy relative to subject-level validation in every model, and between-subject variability was roughly five times the range of per-subject mean performance across the five supervised models.

**What are the implications of the main findings?**

- Feature dimensionality can be cut by 90% and sampling rate reduced to 10–15 Hz with little accuracy loss under the conditions studied, lowering the communication and computation cost of collar-based behavior monitoring.
- Where accuracy cannot separate models, selection should rest on operational properties such as inference cost, transmitted data volume, and the retraining required when the label set changes.

**Abstract:**

Comparisons of behavior classification pipelines rarely vary one design factor at a time or report computational cost. Six-axis inertial measurement unit data were recorded at 25 Hz from collar-mounted sensors on 19 Korean black goats, with labels from a semi-automatic procedure verified against synchronized video. Six models classifying rumination, eating, and other behavior were compared on identical 10 s windows, varying feature source, classifier, extractor architecture, and training objective one at a time, under leave-one-subject-out (LOSO) and stratified 5-fold cross-validation (CV). A 24-dimensional learned representation matched a 250-dimensional handcrafted feature set (Macro F1 0.810 versus 0.814 under LOSO, 0.876 versus 0.876 under 5-fold CV), and two structurally unrelated extractors reached this parity. Replacing the supervised training objective with unsupervised reconstruction reduced Macro F1 by 0.094–0.127. Window-level estimates exceeded LOSO estimates by 0.044–0.095, and between-subject variability was roughly five times the spread among the five supervised models. Sampling at 10–15 Hz retained most of the accuracy at 40–60% lower data volume, and the learned pipelines cut inference time by up to 36-fold in this implementation. These operating points apply to sustained behavioral episodes under the conditions studied; where accuracy cannot separate models, computational cost should govern the choice.

## 1. Introduction

Continuous monitoring of animal behavior is a central goal of precision livestock farming. The time budgets of key behaviors provide information on animal health and welfare that intermittent human observation captures only coarsely; continuous sensing can detect changes earlier and at finer temporal resolution [1]. In ruminants, rumination and eating time are among the most informative behavioral indicators for health surveillance in dairy systems and for health and reproductive monitoring in beef cattle [2,3]. Changes in these behaviors can occur during stress, such as heat stress, and may precede clinically apparent disease [2]. Direct visual observation is labor-intensive, difficult to apply to large groups, and impractical at night or on pasture. These limitations have motivated automated sensor-based monitoring [4]. Goats are economically important in many regions and differ from cattle in body mass, feeding style, and movement dynamics. Parameters developed for cattle therefore need to be validated before they are applied to goats [5,6].

Among wearable sensing technologies, neck-mounted accelerometers and inertial measurement units (IMUs) are the dominant modality for ruminant behavior recognition [6]. Conventional approaches typically combine handcrafted features with classical machine-learning classifiers. Statistical and spectral features are extracted from signal windows and, often after feature selection, classified with decision trees or tree ensembles such as random forests, AdaBoost, or gradient boosting [7,8,9,10]. One example is the calf behavior classification pipeline developed in Kim et al. [11], which reduced its candidate feature set to 250 by first removing highly correlated features and then eliminating those of low tree-based importance, followed by classification with Light Gradient Boosting Machine (LightGBM). Practical deployment, however, involves additional constraints. Collar-mounted systems may need to operate continuously, transmit data over bandwidth-limited wireless links, and monitor large numbers of animals. Classification accuracy must therefore be considered together with feature dimensionality, sampling rate, and computational cost [6,12].

Deep learning provides an alternative in which representations are learned directly from raw signals. A growing number of deep-learning architectures have been applied to livestock behavior classification, but most have been evaluated on different datasets [13]. This makes direct comparison difficult. Studies often differ simultaneously in species, window length, classifier, and validation scheme, so differences in performance cannot easily be attributed to a single design choice [6,14]. It therefore remains unclear whether reported improvements result from the learned representation, the downstream classifier, or the training objective. It is also unclear whether a compact learned representation can perform as well as a high-dimensional handcrafted feature set.

A second issue is how model performance is evaluated. Many studies use cross-validation in which signal windows are assigned to partitions without considering animal identity. Windows from the same animal, and often from the same behavioral bout, may therefore appear in both the training and held-out sets [15]. Because adjacent windows are highly correlated and differences between animals are the main source of variability in behavior classification, such estimates cannot be used to infer generalization to unseen animals [15,16]. Subject-level validation, such as leave-one-subject-out cross-validation, estimates this generalization directly. Rahman et al. [16] compared within-animal and across-animal partitioning on the same cattle dataset and reported a marked reduction in accuracy under the latter. Since then, however, subject-level validation has remained less common, and few studies have reported both schemes on the same dataset [17,18].

To address these gaps, this study compared six models for classifying rumination, eating, and other behaviors in Korean black goats (*n* = 19) wearing a neck-mounted six-axis IMU. Ground-truth labels were produced by a semi-automatic procedure combining manual video annotation with automatic labeling, verified on a stratified sample against synchronized video. The models were evaluated on the same data using both subject-level and window-level validation. We also compared feature source, classifier, extractor architecture, and training objective and evaluated practical factors including sampling rate, inference latency, and transmitted payload. The results show that a 24-dimensional learned representation can match a 250-dimensional handcrafted feature set and that among the models examined, representation quality depends more strongly on the training objective than on the extractor architecture.

## 2. Materials and Methods

### 2.1. Animals and Sensor Data Acquisition

This study was conducted with the approval of the Animal Experimentation Ethics Committee (SNU-250112-3). A total of 19 Korean black goats (*Capra hircus*), all wethers (castrated males) raised for fattening, formed the data acquisition cohort. The cohort comprised 9 animals aged 4–5 months (body weight 28.9 ± 5.2 kg) and 10 aged 6–7 months (40.1 ± 2.4 kg), with an overall mean body weight of 34.8 ± 6.9 kg. Animals were housed in a roofed goat house under standard fattening husbandry and allocated to 2.5 m × 1.6 m floor pens, with two individuals per pen, except one pen that housed a single animal. Each pen contained one water trough and one feed trough, and bedding was replenished as required. A commercial dry total mixed ration formulated for fattening goats (Jinmiseonchan, Cheongsan Co., Chungju, Republic of Korea) and clean water were provided ad libitum throughout. No behavioral manipulation was applied during acquisition, so the recordings reflect spontaneous behavior under conventional husbandry.

**Figure 1:**
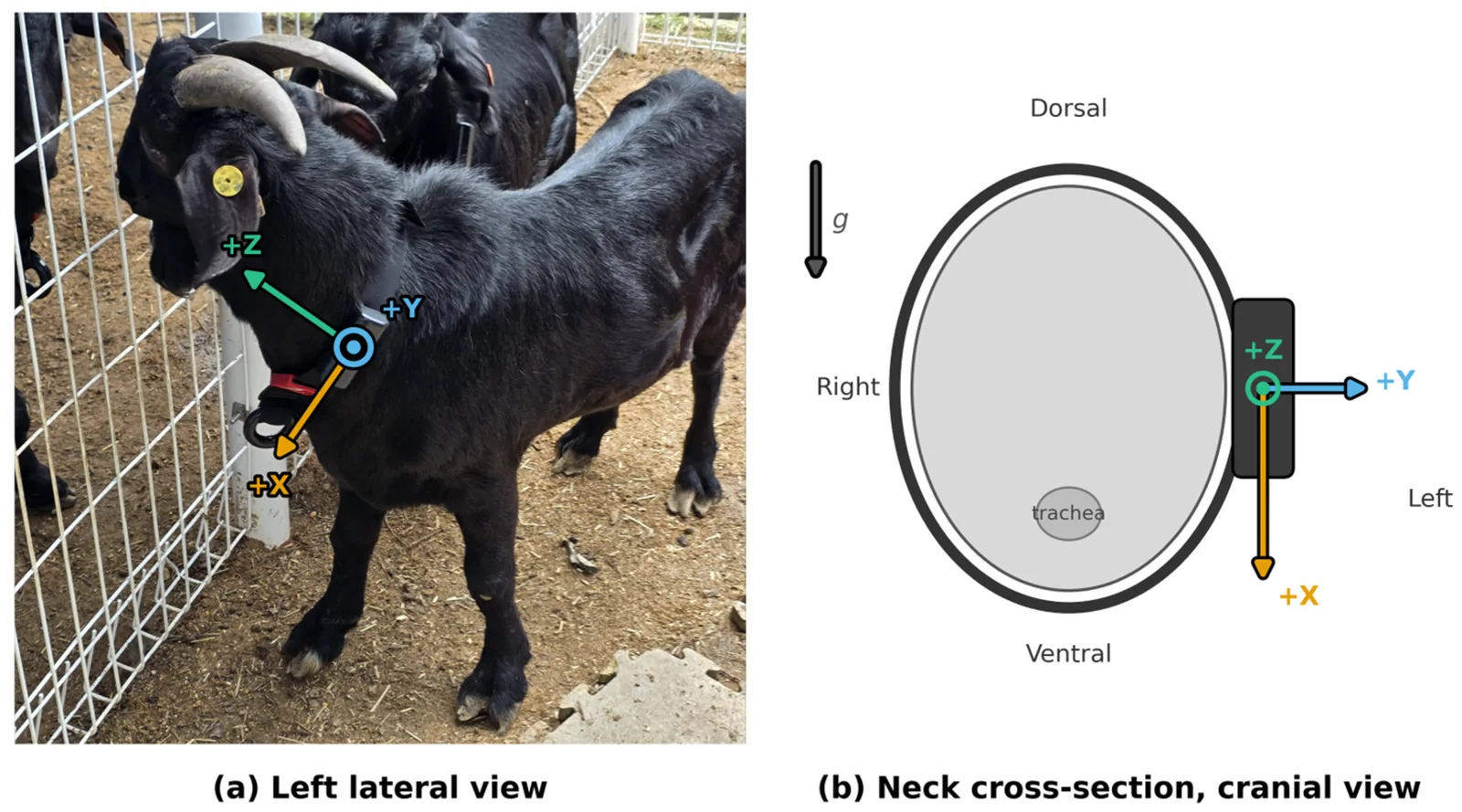
Wearing position of the collar-mounted six-axis IMU on a Korean black goat and orientation of the sensor axes relative to the body: (**a**) left lateral view; (**b**) cross-section of the neck at the collar, cranial view. Arrows indicate the positive axis directions; ⊙ indicates an axis pointing toward the viewer.

Each subject wore a collar-mounted six-axis inertial measurement unit (FH Sensor, Bodit Co., Ltd., Seoul, Republic of Korea). The wearing position and the axis orientation relative to the animal’s body are shown in Figure 1. The sensor provides a three-axis linear accelerometer (channels ax, ay, az; full-scale range ±8 g) and a three-axis rate gyroscope (channels gx, gy, gz; ±1000 °/s), giving six simultaneously sampled channels at a fixed rate of 25 Hz. The raw six-channel time series as delivered by the logger served as the model input in every downstream experiment. Continuous acquisition ran for nine consecutive days, from 18 to 26 September 2025.

The X axis ran along the collar strap (positive ventrally), the Y axis was normal to the neck surface (positive outward), and the Z axis ran along the neck (positive cranially). Collar rotation between individuals and micro-drift during movement introduced subject-specific orientation offsets, which were not quantified. No per-subject orientation calibration was applied. All downstream models were trained and evaluated on raw axis-native signals, which places the burden of orientation invariance on the model and not on a preprocessing pipeline [19].

### 2.2. Reference Video and Ground-Truth Annotation

Reference video was captured by ceiling-mounted internet protocol cameras (Roboview 24-3, Global I-net, Gwangmyeong, Republic of Korea; 1920 × 1080, approximately 30 fps), one camera per pen, covering the full floor area with no blind zones. Video and IMU timelines were aligned manually at the start of each 30 min recording session. IMU timestamps were generated on the logger with a per-sample sequence number and were therefore unaffected by transmission latency. No clock-drift correction was applied; because alignment was repeated every 30 min, accumulated drift remained below 0.2 s even for a 100 ppm oscillator, under 2% of a 10 s window.

Ground-truth labels for the two focal behaviors, rumination and eating, were established through a semi-automatic annotation procedure. Five trained observers, each responsible for a distinct subset of animals with no overlap, first annotated rumination and eating episodes from the reference video using the BoditToolBox video annotation software (version 1.0, Bodit Co., Ltd., Seoul, Republic of Korea), an interval-marking tool; the IMU waveform was not shown to the observers. This manual stage covered 14 animals over 32 animal-days and yielded 43,119 labeled rumination and eating windows. A two-stage automatic labeler was then applied to the full recordings (1,416,961 windows over 164 animal-days). A binary deep-learning model operating on the raw six-channel IMU window first separated the two focal behaviors from all other states, passing windows with P(other) ≤ 0.50 (427,231 windows). A *k*-nearest neighbor (KNN) classifier (*k* = 5, Euclidean distance, uniform weights, z-score normalization) operating on 901 standardized time- and frequency-domain features drawn from the same 903-feature candidate family used by Model A then distinguished rumination from eating. For each window, the classifier reported a confidence score equal to the fraction of the k neighbors agreeing on the predicted class, and only windows with unanimous agreement (360,336 windows) were retained. The binary screening model was developed by the sensor manufacturer, and its architecture is not disclosed here. Because the KNN stage uses the same handcrafted feature family as Model A, the labels are not fully independent of Model A’s input representation. Of the 132,887 windows in the final dataset, 78,320 (58.9%), including Other windows, originated directly from manual annotation and 54,567 (41.1%) from the automatic labeler.

Labeling reliability was assessed on a class-stratified random sample of retained windows (200 per class, 600 in total), which was re-examined against the synchronized reference video by a trained observer. Agreement between the assigned labels and the human video assessment was 99.3% overall (596/600; Wilson 95% CI 98.3–99.7%): Rumination 98.5% (95.7–99.5%), Eating 99.5% (97.2–99.9%), and Other 100.0% (98.1–100.0%). Because each animal was annotated by a single observer, inter-rater reliability could not be computed.

In both the manual annotation and the verification step, observers identified rumination and eating episodes as continuous intervals on the video timeline, using the definitions of rumination and feeding (here termed eating) given by Kim et al. [11]. Only episodes lasting at least 180 s were retained as valid events; very short bouts have likewise been removed in beef bulls, in which they were poorly represented by window-level features [3]. Shorter episodes were excluded from the focal classes to suppress transient misclassifications at behavior boundaries. This criterion also limits the influence of any residual timeline misalignment, since an offset of a few seconds affects at most the first and last 10 s window of each episode.

### 2.3. Dataset Construction

Interval-level annotations were mapped onto the IMU sample timeline and partitioned into contiguous, non-overlapping 10 s windows (250 samples × 6 channels). Window length affects classification accuracy [20]; 10 s is the median window length among accelerometer studies of ruminant behavior [6] and has been used in dairy cows [7] and beef calves [11]. The window is the atomic unit for model training and evaluation. Non-overlapping windows were used, whereas Kim et al. [11] used 50% overlap; this avoids sharing samples between adjacent windows and therefore between cross-validation partitions. Windows containing more than one behavior class were excluded, so each retained window carries a single unambiguous label; studies in beef bulls [3] and beef calves [11] instead assigned such windows to the prevailing behavior. Together with the 180 s minimum-duration criterion, this rule removes ambiguous windows at behavior transitions.

The Other class covers behavioral states outside rumination and eating. It was drawn mainly from the subset of (subject, day) recordings for which annotation covered the full 24 h period, so that intervals containing neither focal behavior could be identified unambiguously; these intervals were partitioned into 10 s windows by the same procedure used for the two target classes. Seven fully annotated animal-days from six animals contributed the large majority of Other windows, with the remainder drawn from unlabeled intervals of partially annotated animal-days. Rumination and eating episodes shorter than 180 s were not retained as focal events, and the corresponding intervals were therefore treated as Other. Windows of all three classes were then randomly subsampled without replacement (NumPy RandomState, seed = 42) to a comparable per-class count, approximately 44,000 each, giving an aggregate ratio of about 1:1:1. The retained per-class counts therefore reflect this balancing and the high-confidence criteria, not the total time each behavior occupied during recording. The numbers of windows per subject and class are given in Table S4. The final dataset composition is summarized in Table 1.

### 2.4. Model Design

#### 2.4.1. Design Rationale

**Figure 2:**
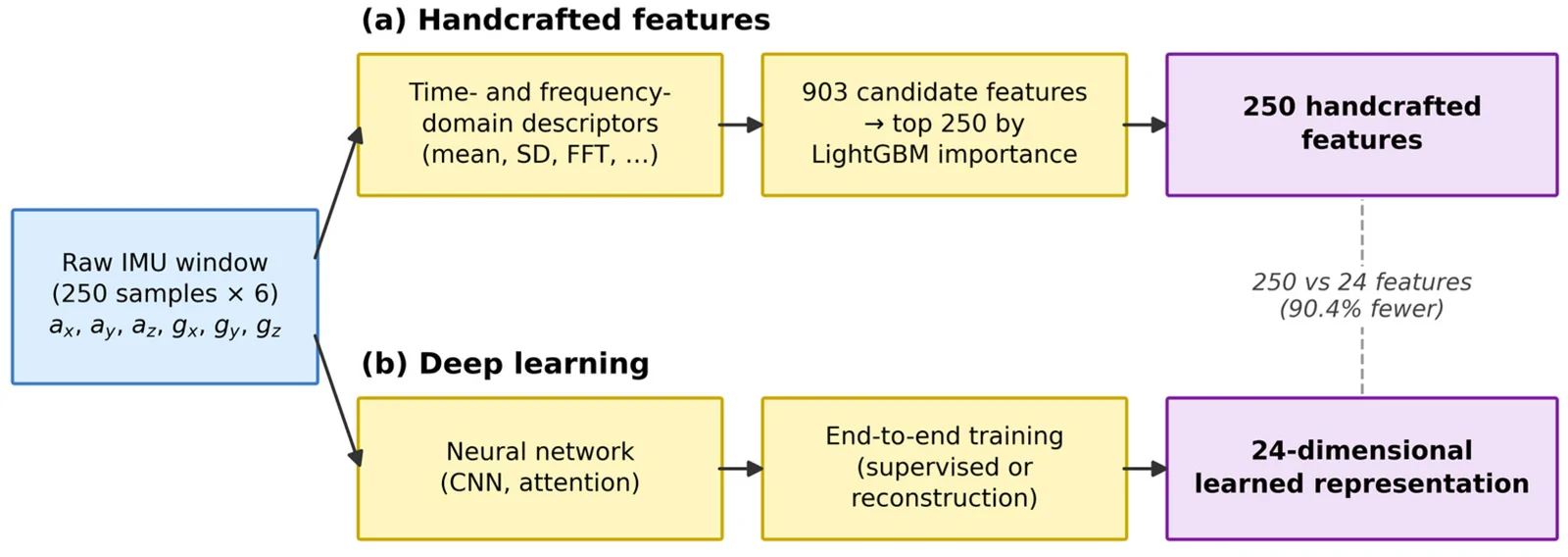
The two feature extraction paradigms compared in this study. (**a**) Handcrafted: raw IMU signal → statistical and spectral analysis → 903 candidate features → top 250 selected by feature importance. (**b**) Deep learning: raw IMU signal → neural network trained end-to-end → 24-dimensional learned representation.

Six models (A–F) were designed for a controlled comparison of feature extraction paradigms and downstream classifiers on identical data (Figure 2). Model A reproduced the handcrafted feature pipeline of Kim et al. [11] and served as the baseline. Models B and C shared the same learned 24-dimensional feature extractor and differed only in the downstream classifier: LightGBM (Model B) or KNN (Model C). Model D was an end-to-end alternative in which feature extraction and classification were integrated in a single network. Models E and F were ablation variants that isolated the contribution of the extractor architecture (an attention-based versus multi-scale convolutional neural network, or CNN) and of the training signal (supervised versus unsupervised). All six operated on the same 250 × 6 input tensor. Models A, B, E, and F shared an identical downstream LightGBM classifier, so differences in classification performance were attributable to the feature extraction stage and not to classifier tuning.

#### 2.4.2. Model A: Handcrafted Features + LightGBM

Model A reproduced the handcrafted feature pipeline of Kim et al. [11] on the goat dataset. In the present reimplementation, 903 candidate features were computed from each 10 s × 6-channel window. These comprised time-domain descriptors (per-channel mean, standard deviation, skewness, kurtosis, interquartile range, and zero-crossing rate) and frequency-domain descriptors (fast Fourier transform coefficients, spectral energy, and band powers), evaluated both per axis and on selected cross-axis combinations. The top 250 features were retained by LightGBM feature importance ranking. This reproduced the feature dimensionality used by Kim et al. [11], which reached 250 features in two stages by first discarding highly correlated candidates; the present study applied importance ranking alone. The retained features were passed to a LightGBM classifier [21].

#### 2.4.3. Multi-Level Attention Feature Extractor (Models B and C)

**Figure 3:**
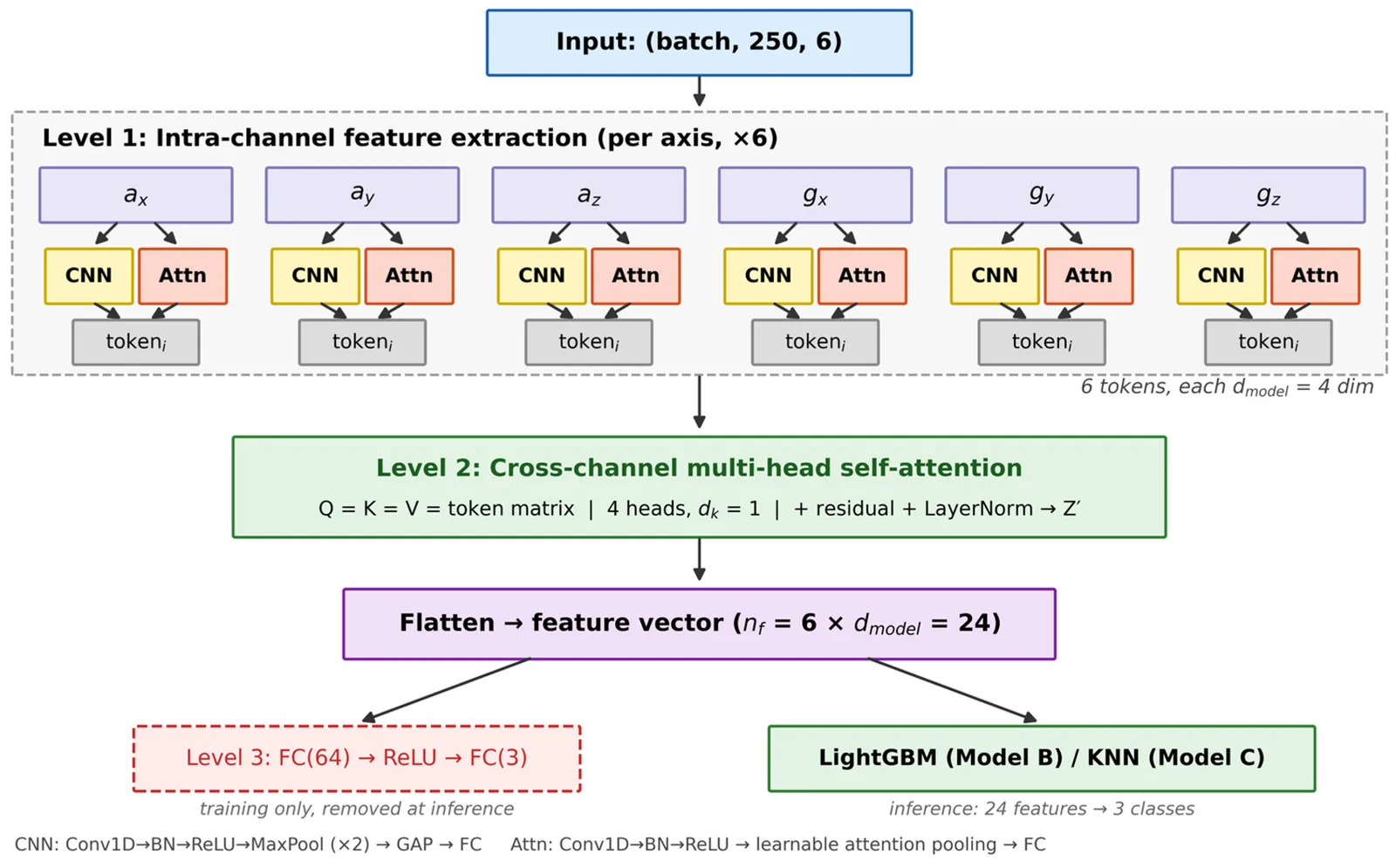
Architecture of the multi-level attention feature extractor used in Models B and C.

Models B (LightGBM downstream classifier) and C (KNN downstream classifier) shared a hierarchical three-level feature extractor that mapped a raw IMU window of size 250 × 6 to a 24-dimensional learned representation (Figure 3).

Level 1—Intra-channel feature extraction: Each of the six IMU channels, a length-250 univariate sequence, is processed independently by a parallel CNN branch and attention branch. The CNN branch extracts local temporal patterns through two stacked Conv1D → batch normalization → rectified linear unit (ReLU) → max-pooling blocks (kernel sizes 7 and 5, output channels 16 and 32), followed by global average pooling (GAP) and a fully connected (FC) projection to dimension *d*.

$$CNN(x_{i})={FC}_{32\to d}\left(GAP\left({Conv}_{16\to 32}^{k=5}\left({Conv}_{1\to 16}^{k=7}(x_{i})\right)\right)\right)$$

The attention branch first embeds the channel into a length-250 sequence of 32-dimensional feature vectors via Conv1D (kernel size 7) → batch normalization (BN) → ReLU.

$$h=ReLU\left(BN\left({Conv}_{1\to 32}^{k=7}(x_{i})\right)\right)\in R^{T\times 32}$$

A learnable scoring vector then assigns an importance score to each time step, and a softmax over time produces attention weights *α_t_*.

$$\alpha_{t}=\frac{exp(w^{⊤}h_{t})}{\sum_{\tau =1}^{T}exp(w^{⊤}h_{\tau })}$$

The attention output is the weighted temporal sum of embeddings, followed by an FC projection to dimension *d*.

$$Attn(x_{i})={FC}_{32\to d}\left(\sum_{t=1}^{T}\alpha_{t}\cdot h_{t}\right)$$

The CNN and attention outputs are concatenated to form a per-channel token of dimension *d*_model_ = 2*d*.

(1) $$z_{i}=[\;CNN(x_{i})\;;\;Attn(x_{i})\;]\in R^{2d}$$

Level 2—Cross-channel self-attention: The six per-channel tokens are stacked into a 6 × *d*_model_ matrix ***Z*** and passed through a standard multi-head self-attention (MHA) layer [22],

$$MHA(Z)=softmax\left(\frac{ZW_{Q}(ZW_{K}{)}^{⊤}}{\sqrt{d_{k}}}\right)ZW_{V},$$

followed by a residual connection, dropout, and layer normalization, giving the output ***Z′***.

$$Z^{\prime }=LayerNorm\left(Z+Dropout(MHA(Z))\right)$$

The number of attention heads is set to the largest divisor of *d*_model_ not exceeding four; with *d*_model_ = 4 this gives four heads of dimension *d_k_* = 1.

Level 3—Classification head: For end-to-end training, ***Z′*** is flattened to a vector of length *n_f_* = 6 × *d*_model_ = 6 × 2*d*. A two-layer classification head (FC → ReLU → FC, hidden width 64) then produces class logits over the three behavior classes.

$$\hat{y}={FC}_{64\to 3}\left(ReLU({FC}_{n_{f}\to 64}(flatten(Z^{\prime })))\right)$$

At inference time, the classification head is discarded, and the flattened representation is used as the feature vector supplied to the downstream classifier.

The latent dimension is set to *d* = 2, giving *d*_model_ = 4, *n_f_* = 24, and approximately 21 K trainable parameters. This is the smallest configuration in the architectural family, chosen to test whether a much more compact representation could match the 250-dimensional handcrafted baseline. A pilot comparison of *d* = 2, 4, and 8 (24, 48, and 96 features), run on an earlier version of the dataset with a different validation protocol and a KNN classifier, gave Macro F1 of 0.772, 0.777, and 0.779; these values are not directly comparable to the main experiments. The results below therefore show that 24 dimensions suffice for this task, not that 24 is optimal.

The 24-dimensional representation is passed to a downstream classifier that differs between the two models: LightGBM in Model B and KNN (*k* = 5, Euclidean distance) in Model C. Model C tests whether the representation retains discriminative structure under a non-parametric classifier that applies no additional feature weighting.

#### 2.4.4. Temporal Attention-CNN End-to-End Network (Model D)

Model D integrates feature extraction and classification into a single network that takes the raw IMU window ***x*** ∈ ℝ^250×6^ as input and directly outputs class logits over the three behavior classes, without any external classifier.

The input is first projected linearly from 6 channels to *d*_model_ dimensions (the model dimension of Model D, *d*_model_ = 24), and a learnable positional encoding ***P***_pos_ is added to preserve temporal ordering.

$$H_{0}={Linear}_{6\to d_{\mathrm{model}}}(x)+P_{pos}$$

The resulting sequence is passed through a 2-layer Transformer encoder using Gaussian error linear unit (GELU) activation and a feedforward dimension of 2 × *d*_model_, which models temporal and inter-channel correlations. The encoder output is then processed by a 3-layer 1-D CNN with kernel sizes 7, 5, and 3, followed by global average pooling to produce a *d*_model_-dimensional summary vector. A final fully connected layer maps this vector to the three-class logits.

The total number of trainable parameters is approximately 32 K.

#### 2.4.5. Multi-Scale CNN Ablation Variants (Models E and F)

Models E and F use a multi-scale CNN feature extractor without attention, combined with inter-channel Pearson correlation coefficients as auxiliary features. Three parallel CNN branches with kernel sizes 3, 7, and 15 capture temporal patterns at different scales. Each branch consists of two stacked Conv1D → BN → ReLU layers followed by global average pooling, producing a 32-dimensional output per branch and 96 dimensions in total.

Pearson correlation coefficients are computed between every pair of the six IMU channels, yielding C(6, 2) = 15 coefficients.

$$\rho_{ij}=\frac{\sum_{t}\left(x_{i}^{t}-{\bar{x}}_{i}\right)\left(x_{j}^{t}-{\bar{x}}_{j}\right)}{\sqrt{\sum_{t}{\left(x_{i}^{t}-{\bar{x}}_{i}\right)}^{2}\cdot \sum_{t}{\left(x_{j}^{t}-{\bar{x}}_{j}\right)}^{2}}}$$

The 96-dimensional CNN output and the 15-dimensional correlation vector are concatenated into a 111-dimensional representation, which a fully connected layer then projects to the final 24-dimensional feature vector.

Model E is trained in a supervised manner with Focal Loss, and the resulting 24-dimensional feature vector is passed to a LightGBM classifier. Model F replaces supervised training with an unsupervised autoencoder objective [23]. The 24-dimensional bottleneck output is fed to a decoder of ConvTranspose1D layers that reconstructs the original six-channel input, and the network is trained end-to-end with mean squared error (MSE) reconstruction loss. At inference, the encoder is frozen, and its 24-dimensional bottleneck output is passed to a LightGBM classifier trained separately on the labeled data.

#### 2.4.6. Model Summary

Table 2 summarizes the six models across their feature extractor, downstream classifier, feature dimensionality, number of trainable parameters, and estimated model size on disk.

**Figure 4:**
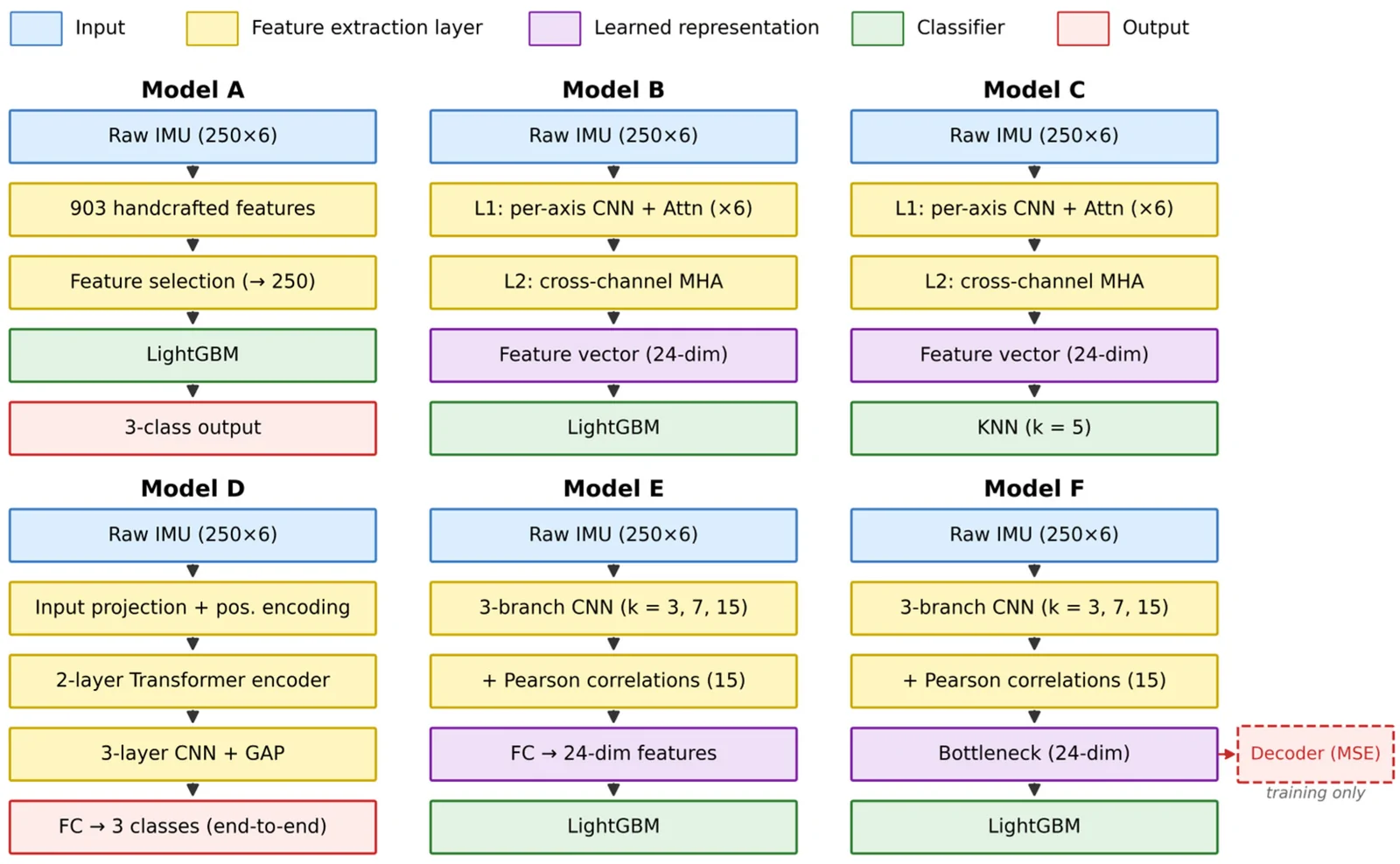
The six model architectures, each shown as a vertical pipeline from raw IMU input to classification output. Block colors indicate input, feature extraction, learned representation, classifier, and output stages.

The six architectures are compared in Figure 4.

### 2.5. Downstream Classifier Configuration

All models except Model D use an external downstream classifier: LightGBM for Models A, B, E, and F, and KNN for Model C. LightGBM was retained because Model A reproduces the pipeline of Kim et al. [11], in which it was the final classifier. With about 133,000 windows, and with row and column subsampling (bagging_fraction = 0.8, feature_fraction = 0.8) acting as regularization, over-fitting of leaf-wise tree growth is limited. The hyperparameters of both classifiers were fixed a priori (Table 3) and not tuned for any model, because tuning the classifier separately for each feature representation would confound the feature-extraction contrast that the design is meant to isolate.

The KNN classifier of Model C uses *k* = 5, Euclidean distance, and StandardScaler normalization of the 24-dimensional feature vector, fitted on the training partition of each split.

### 2.6. Model Training Protocol

All supervised deep learning feature extractors (Models B, C, D, and E) are trained with focal loss [24] to emphasize harder examples and to accommodate the residual class imbalance present within individual training partitions:

$$FL(p_{t})=-\alpha_{t}(1-p_{t}{)}^{\gamma }log(p_{t}),\;\gamma =2.0$$

where *α_t_* is a class-weighting factor set to the inverse class frequency computed on the training partition.

Optimization uses AdamW with an initial learning rate of 1 × 10^−3^ and weight decay 1 × 10^−4^ [25]. The learning rate follows a linear warmup over the first 5 epochs and then decays on a cosine annealing schedule [26]. Training stops when the validation loss does not improve for 15 consecutive epochs. The validation loss is computed on data disjoint from the batches used for parameter updates. Batch size is 256, and gradients are clipped to a maximum L2 norm of 1.0. Input signals are z-score-normalized per channel using statistics computed on the training partition.

The neural networks of Models B–F all follow this protocol, except that Model F is trained with mean squared error reconstruction loss instead of focal loss, consistent with its unsupervised autoencoder objective.

### 2.7. Validation Strategy

**Figure 5:**
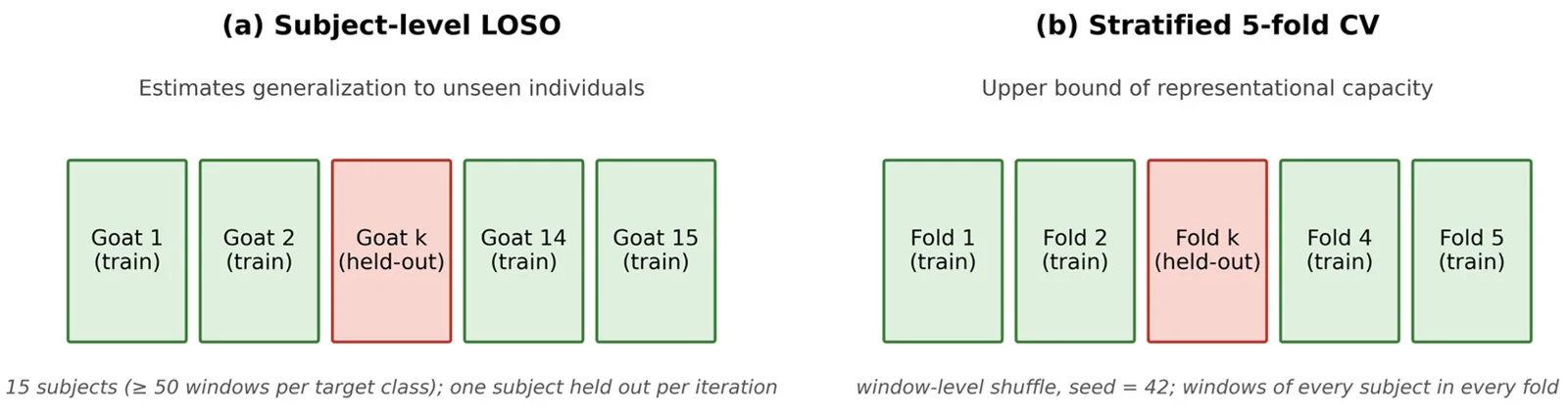
The two validation schemes. (**a**) Subject-level leave-one-subject-out validation over the 15 subjects with at least 50 windows per target class, which is the primary scheme, and (**b**) stratified 5-fold cross-validation with window-level shuffling (seed = 42), interpreted as an upper bound of representational capacity.

Model performance was evaluated using two complementary validation schemes (Figure 5).

Leave-one-subject-out (LOSO) validation holds out all data from a single subject as the validation partition and trains on the remaining subjects, cycling through all subjects. To ensure sufficient per-class support in the held-out partition, LOSO was restricted to the 15 subjects with at least 50 windows in both the Rumination and Eating classes; because the Other class came mainly from seven fully annotated animal-days, seven of these subjects contributed no Other windows. LOSO, a subject-based partitioning method (Kim et al. [11] likewise separated training and held-out data by individual, with a single hold-out of six calves), has no subject-level leakage and estimates how the training procedure generalizes to unseen individuals; it does not evaluate a single fixed model on new animals, and its estimates are comparisons among models under matched conditions, not field accuracy. As the appropriate scheme for assessing predictive performance on new animals, LOSO is treated as the primary result.

Stratified 5-fold cross-validation (5-fold CV) partitions the full dataset into 5 folds while preserving the class distribution in each (scikit-learn StratifiedKFold, shuffle = True, random_state = 42). Windows are assigned to folds independently of subject and of temporal position, so windows from the same animal and consecutive windows from the same behavioral bout can appear in both partitions. Such estimates cannot be used to infer generalization to unseen animals; they estimate an upper bound on the representational capacity of each model under within-subject sampling. The scheme is retained because it remains widely used in this area of the literature and provides a reference point for published figures obtained under comparable partitioning.

The primary evaluation metric is Macro F1, the unweighted mean of the per-class F1 scores; per-class F1, precision, and recall are also reported. For LOSO, two statistics are reported, because they weight subjects differently: the aggregate Macro F1 (computed on predictions concatenated across all held-out subjects) and the mean ± SD of per-subject Macro F1 (*n* = 15) (where each subject’s Macro F1 is averaged over the classes present in its held-out partition).

### 2.8. Sampling Rate Robustness Evaluation

To evaluate model robustness to reduced temporal resolution, the original 25 Hz IMU signals are downsampled to 20, 15, 10, and 5 Hz using the Fourier-based scipy.signal.resample function [27]. All six models are independently re-trained and evaluated at each sampling rate, with the same training protocol and validation schemes as the 25 Hz experiments.

For Model A, the handcrafted pipeline is re-executed at each sampling rate. Two considerations apply. At lower rates, features that depend on frequency content above the corresponding Nyquist limit, for example band power above 2.5 Hz at 5 Hz, are either undefined or aliased, and such out-of-band features are set to zero. The top-250 feature selection is also performed once, on the 25 Hz goat data, and the same 250 indices are reused at all lower rates. This preserves direct comparability with the baseline pipeline of Kim et al. [11], at the cost of not re-selecting rate-specific features.

For Models B, C, D, E, and F, the resampled raw signals are passed directly to the feature extractor. Variable input length across sampling rates is handled by an AdaptiveAvgPool1D layer in Models B, C, E, and F and by the learnable positional encoding in Model D.

### 2.9. Inference Latency Measurement

Inference latency is measured for Models A, B, and D. Model A serves as the baseline, and Models B and D represent the modular and end-to-end designs.

All latency measurements use batch size = 1, corresponding to single-window inference at deployment time. For each model–sampling rate combination, 10 warm-up iterations are executed before 50 timed iterations, and the median of the 50 timed measurements is reported. PyTorch models are evaluated under model.eval() and torch.no_grad(). For GPU measurements, torch.cuda.synchronize() is called at the start and end of each timed iteration to correctly include asynchronous kernel execution time. For CPU measurements, execution is restricted to a single thread via torch.set_num_threads(1).

The measurement scope differs by model according to its inference pipeline. Model A latency covers handcrafted feature extraction and LightGBM prediction. Model B latency covers the deep-learning feature extractor forward pass and LightGBM prediction, the latter contributing 0.12–0.18 ms. Model D latency covers the end-to-end network forward pass with no external classifier. Sensor data acquisition, network transmission, and disk I/O are excluded from all measurements.

The handcrafted feature pipeline of Model A is implemented in Python/NumPy without compiled-language optimization, whereas Models B and D execute through PyTorch operators backed by optimized compiled kernels. Reported latencies therefore reflect these specific implementations rather than an intrinsic property of the two feature-extraction paradigms.

All latency measurements were performed on the workstation described in Section 2.10 and are reported as CPU single-thread and GPU (RTX 3090) values. They characterize relative computational cost across models, not latency on embedded hardware such as ARM Cortex-M-class microcontrollers, which would require on-device profiling.

### 2.10. Reproducibility and Software Environment

All experiments, covering model training, sampling rate evaluation, and inference latency measurement, were performed on a single Linux workstation running Ubuntu 24.04.3 LTS (Canonical Ltd., London, UK), with an Intel x86_64 CPU (Intel Corporation, Santa Clara, CA, USA; 10 physical/20 logical cores, 125.7 GB RAM) and an NVIDIA GeForce RTX 3090 GPU (NVIDIA Corporation, Santa Clara, CA, USA; 24 GB GDDR6X). All models were trained and evaluated in an identical software environment: Python 3.12.13 (Python Software Foundation, Beaverton, OR, USA), PyTorch 2.11.0 [28] with CUDA 13.0 (NVIDIA Corporation), LightGBM 4.6.0 (Microsoft Corporation, Redmond, WA, USA) [21], scikit-learn 1.8.0 [29], NumPy 2.4.4 [30], and SciPy 1.17.1 [27]. All random number generators (PyTorch, NumPy, LightGBM) were seeded with 42, which fixes weight initialization, data ordering, and partition assignment. Deterministic GPU kernel execution was not enforced, so repeated runs of the same configuration may differ in the third decimal place of Macro F1.

### 2.11. Statistical Analysis

Models were compared on per-subject Macro F1 under LOSO at 25 Hz, averaged over the classes present in each subject’s held-out data, with each held-out subject as the experimental unit (*n* = 15). A linear mixed model was fitted with model (A–F) as a fixed effect and subject as a random intercept, using R version 4.6.1 (R Foundation for Statistical Computing, Vienna, Austria) [31] and the lme4 package (version 2.0-6) [32]. Estimated marginal means and Tukey-adjusted pairwise contrasts with 95% confidence intervals were obtained with the emmeans package [33], and paired Cohen’s *d_z_* is reported as the effect size for the contrasts discussed in the text. Fold-level differences under 5-fold CV were not tested, because folds are resamples of a single dataset rather than independent experimental units. To quantify run-to-run variation, the full LOSO protocol at 25 Hz was repeated with 6 training seeds (42–47), with the dataset and subject partitions unchanged, and the mixed model was refitted to each run.

## 3. Results

### 3.1. Overall Model Comparison

Under subject-level LOSO, Models A, B, D, and E fell within 0.013 of one another in aggregate Macro F1 (0.810–0.823), Model C was lower (0.780), and Model F was lowest (0.685). Model D ranked first by aggregate Macro F1 and Model A first by the mean of per-subject Macro F1 (Table 4). The mixed model indicated a significant overall effect of model (*F*(5, 70) = 13.1, *p* < 0.001), but no pairwise contrast among Models A, B, D, and E reached significance (Tukey-adjusted *p* ≥ 0.97). Estimated differences among these four models were at most 0.008, and every Tukey-adjusted 95% confidence interval included zero (A versus B, 0.006 [−0.027, 0.040], *d_z_* = 0.45; A versus D, 0.006 [−0.027, 0.040], *d_z_* = 0.19; B versus D, 0.000 [−0.033, 0.033], *d_z_* = 0.00). Model C was numerically lower than Models A and B (by 0.025 and 0.019) but not significantly so (*p* = 0.25 and 0.55). Model F was significantly below every other model (differences 0.052–0.078, all *p* < 0.001); no other contrast reached significance. Across six repeated runs with different training seeds, the standard deviation of aggregate LOSO Macro F1 was 0.0005–0.0037 (Table 4 and Table S5), and refitting the mixed model to each run gave the same result: no pairwise contrast among Models A, B, D, and E reached significance (Tukey-adjusted *p* ≥ 0.89), and Model F was below every other model (*p* ≤ 0.001).

Under stratified 5-fold CV, Models A, B, and E formed a top cluster within 0.001 of one another in aggregate Macro F1, and Model F ranked lowest (Table 4). Model B matched Model A (difference 0.0001) using a 24-dimensional learned representation in place of 250 handcrafted features, a 90.4% reduction in feature dimensionality. Model E reached the same range with an attention-free multi-scale CNN extractor, also at 24 dimensions. Models A and B each exceeded Model D in all five folds.

The reduction from 5-fold CV to LOSO in aggregate Macro F1 was itself model-dependent, ranging from 0.044 (Model D) to 0.095 (Model F), with Models A, E, B, and C intermediate at 0.061, 0.062, 0.066, and 0.085. Model D showed the smallest reduction and Model F the largest at every sampling rate (Table S1).

Dispersion differed markedly between the two schemes. Across the five folds, the standard deviation of Macro F1 was 0.0008 to 0.0021 for Models A–E and 0.0079 for Model F. Across the 15 held-out subjects, the standard deviation of per-subject Macro F1 was 0.118 to 0.150 for Models A–E and 0.174 for Model F. Near-zero variation between folds follows from every fold containing windows from every subject, so the five partitions are resamples of a single evaluation problem and not five distinct ones. The full range of per-subject mean Macro F1 across Models A–E was 0.025, smaller than the between-subject standard deviation by a factor of approximately 5.

Table 4 summarizes the classification performance of the six models under both validation schemes.

### 3.2. Per-Class F1 Comparison Across All Six Models

#### 3.2.1. Subject-Level LOSO

All six models produced the same ordering of class difficulty, defined here by the per-class F1 attained across models: Eating highest, Rumination intermediate, and Other lowest. Model D reached the highest LOSO Macro F1 (0.823) and the highest F1 in Rumination (0.828) and Other (0.756), while Model E was highest in Eating (0.886). The between-model spread within each class was 0.040 for Eating, 0.137 for Rumination, and 0.237 for Other.

**Figure 6:**
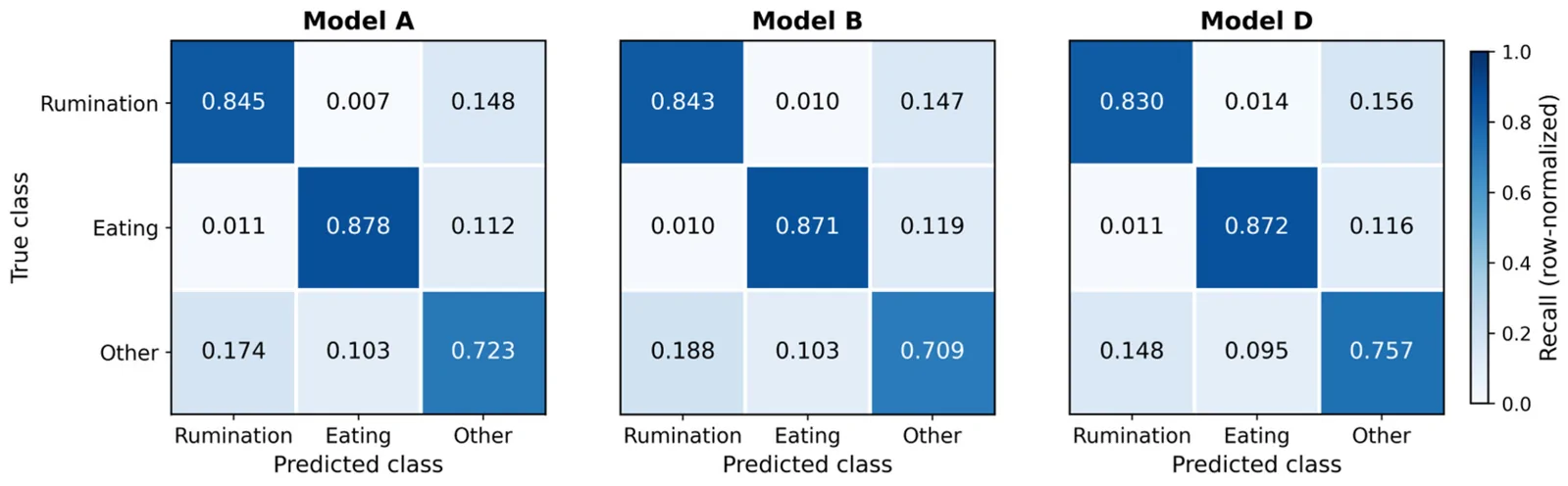
Row-normalized confusion matrices for Models A, B, and D under subject-level LOSO at 25 Hz; cell values are per-class recall. Values derive from an independent re-execution of the protocol.

Figure 6 shows row-normalized confusion matrices for Models A, B, and D under LOSO at 25 Hz. In all three, Other has the lowest diagonal recall and is most often confused with Rumination, while Eating has the highest. These matrices come from an independent re-execution of the LOSO protocol under identical seeds; per-class F1 derived from them differs from Table 5 by at most 0.006, and aggregate Macro F1 by at most 0.003, reflecting non-deterministic GPU execution.

Per-class F1 under LOSO is given in Table 5, and per-class precision and recall in Table S3.

#### 3.2.2. Five-Fold Cross-Validation

The class difficulty ordering was the same as under LOSO. Models A and B were identical to three decimals in every class, and Model E recorded the highest Eating F1 of any model (0.924). The spread between the highest- and lowest-scoring model widened with class difficulty: 0.050 for Eating, 0.094 for Rumination, and 0.148 for Other.

The reduction from 5-fold CV to LOSO followed the same ordering in all six models, being the smallest in Eating and the largest in Other. For Model A, the reductions were 0.034 (Eating), 0.062 (Rumination), and 0.088 (Other). Per-class F1 under 5-fold CV is given in Table 6.

### 3.3. Ablation of Feature Extraction and Classification

To isolate individual design choices, model pairs differing primarily in one variable were compared (Table 7). Model D is excluded because it differs from all other models in both feature extractor and classifier.

Replacing the 250 handcrafted features of Kim et al. [11] with a 24-dimensional learned representation changed Macro F1 by at most 0.005 under either scheme. This held for two structurally unrelated extractors: a multi-level attention network (the A-versus-B contrast) and an attention-free multi-scale CNN (A versus E).

Changing the training objective from supervised to unsupervised reconstruction with extractor and classifier held fixed (E versus F) reduced Macro F1 by 0.127 under LOSO and 0.094 under 5-fold CV. This is more than an order of magnitude larger than either feature extraction contrast.

Replacing LightGBM with KNN at fixed representation (B versus C) reduced Macro F1 by 0.030 under LOSO and 0.011 under 5-fold CV, showing a 2.7-fold larger reduction under the subject-level scheme.

### 3.4. Sampling Rate Robustness

Macro F1 increased monotonically with sampling rate for Models A, B, and E under both schemes and for Model D under LOSO and non-decreasingly for Model C, whose LOSO values were identical at 15 and 20 Hz. The only decrease among Models A–E was Model D under 5-fold CV, which peaked at 20 Hz. Gains were front-loaded. For Models A and B, the 5 to 10 Hz step accounted for more than half of the total 5 to 25 Hz gain, while 20 to 25 Hz contributed no more than 0.003. Across Models A–E, the total 25 to 5 Hz reduction ranged from 0.022 (Model A) to 0.043 (Model C) under 5-fold CV.

The Macro F1 difference between Models A and B widened as sampling rate decreased under 5-fold CV, in favor of Model A, from 0.000 at 25 Hz to 0.009 at 5 Hz; however, it showed no comparable trend under LOSO. Because Model A reused the feature indices selected at 25 Hz, with out-of-Nyquist features set to zero (Section 2.8), its values below 25 Hz are a conservative estimate of what that pipeline can attain.

Model F varied non-monotonically across the five sampling rates within a range of 0.013 under 5-fold CV and 0.010 under LOSO, showing the smallest sampling rate dependence of any model.

Model D showed the smallest gap between 5-fold CV and LOSO Macro F1 at every sampling rate (0.044–0.056), and Models C and F the largest (0.074–0.085 and 0.093–0.115, respectively). Models A, B, and E were intermediate throughout, at 0.060–0.067, and their relative ordering varied across sampling rates.

**Figure 7:**
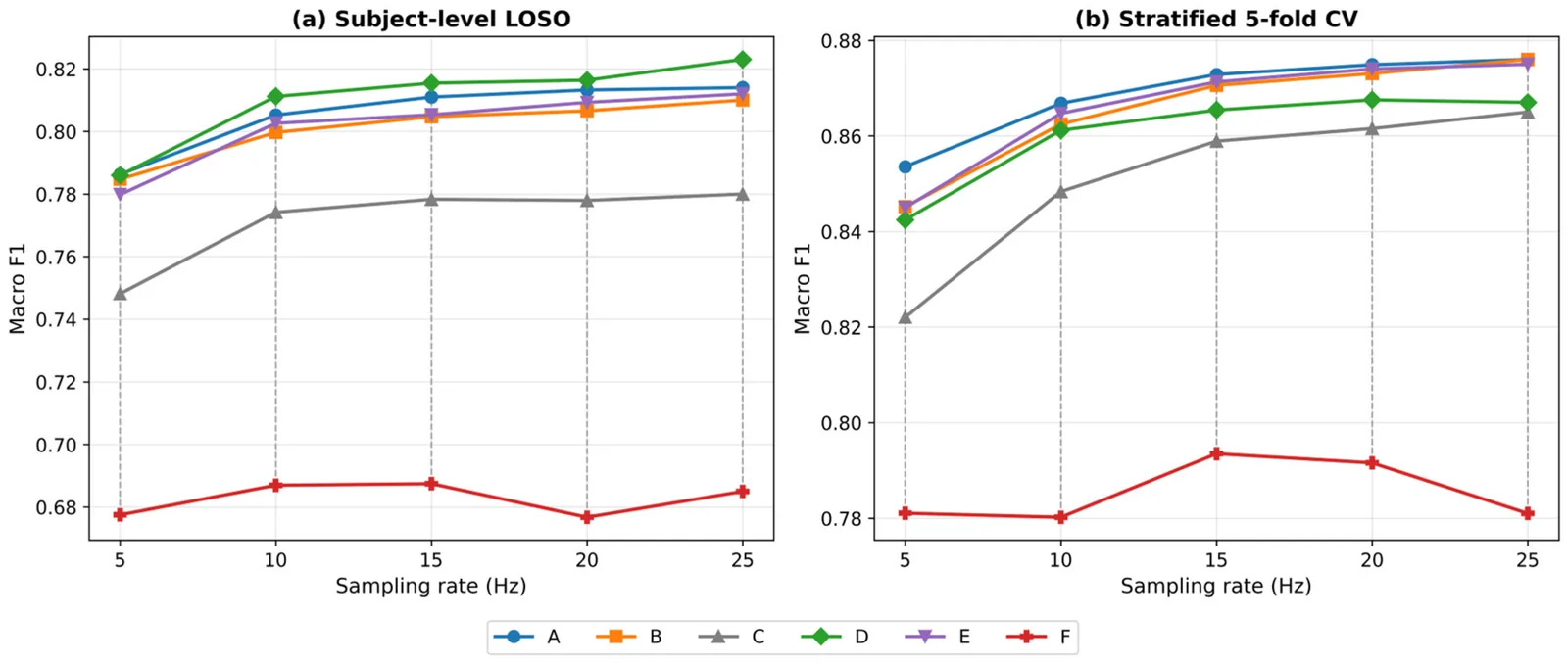
Macro F1 as a function of sampling rate for all six models under (**a**) subject-level LOSO and (**b**) stratified 5-fold cross-validation. Vertical dashed lines mark the inter-model gap (max–min Macro F1) at each sampling rate.

Table S1 and Figure 7 report Macro F1 for all six models at 5, 10, 15, 20, and 25 Hz under both validation schemes; per-class values at all sampling rates are provided in Table S2.

### 3.5. Inference Latency

On CPU, Model A required 24.35 to 43.16 ms per window, Model B 2.15 to 2.65 ms, and Model D 0.68 to 1.55 ms. The same-rate speedups over Model A were 11.3–16.3× for Model B and 26.0–36.1× for Model D. Model A’s measurements were not strictly monotonic with sampling rate, so its scaling should be read only as an overall trend.

The two neural models scaled differently with input length. Over the fivefold increase in sequence length from 5 to 25 Hz, Model B CPU latency increased 1.23-fold, while Model D increased 2.28-fold.

Device ranking was not consistent. Model B was faster on CPU than on GPU at every sampling rate, whereas Model D reversed between 10 and 15 Hz. Its GPU latency varied by only 0.11 ms across the full sampling-rate range.

All measured configurations exceeded the real-time requirement of one 10 s window per 10 s of wall-clock time by two to four orders of magnitude.

**Figure 8:**
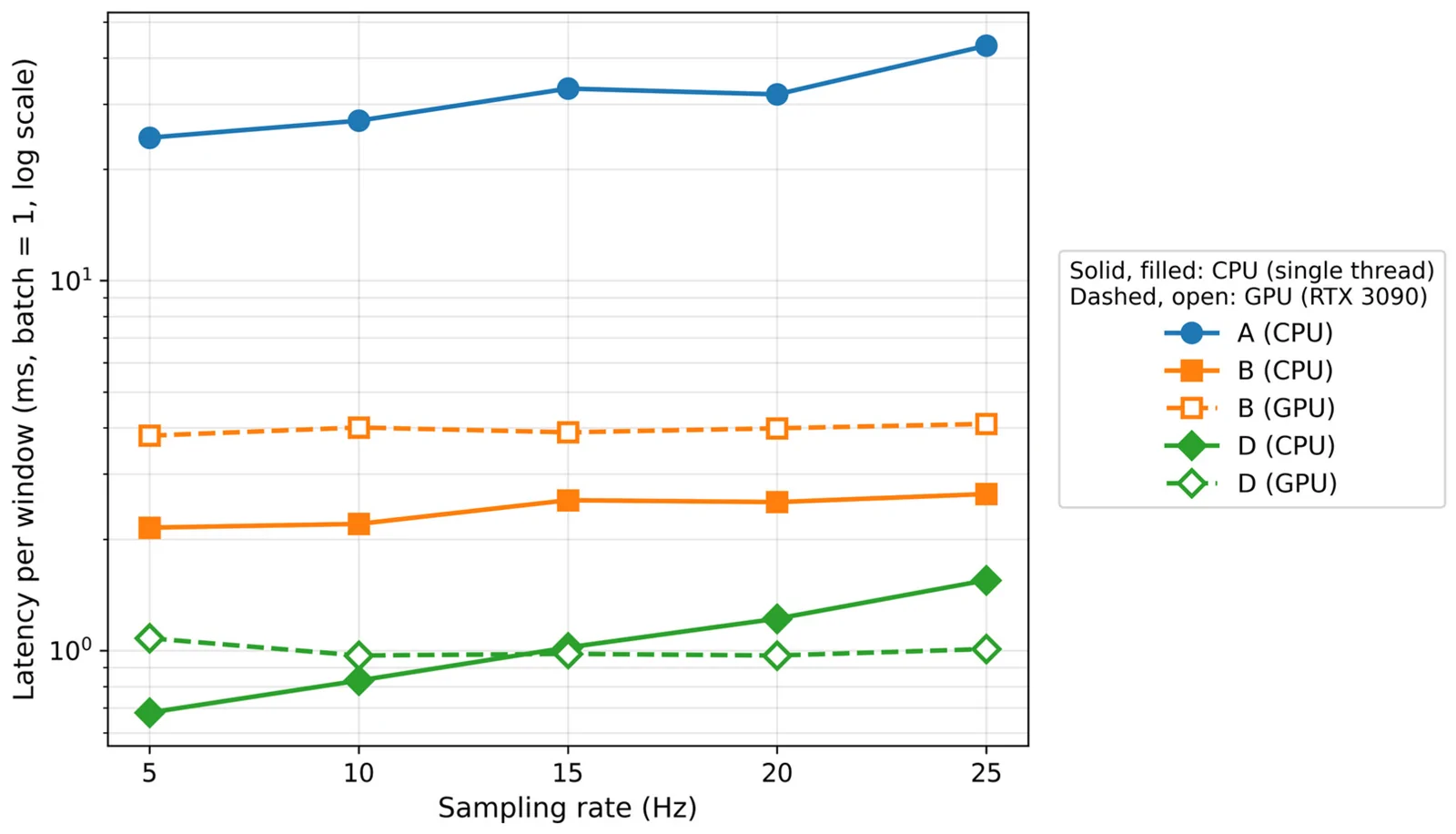
Per-sample inference latency by sampling rate (batch = 1, log scale, median of 50 timed runs after warm-up). Numerical values are given in Table 8.

Per-sample latencies at batch size 1 are given in Table 8 and Figure 8.

### 3.6. Speed–Accuracy Trade-Off and Aggregate Reduction

**Figure 9:**
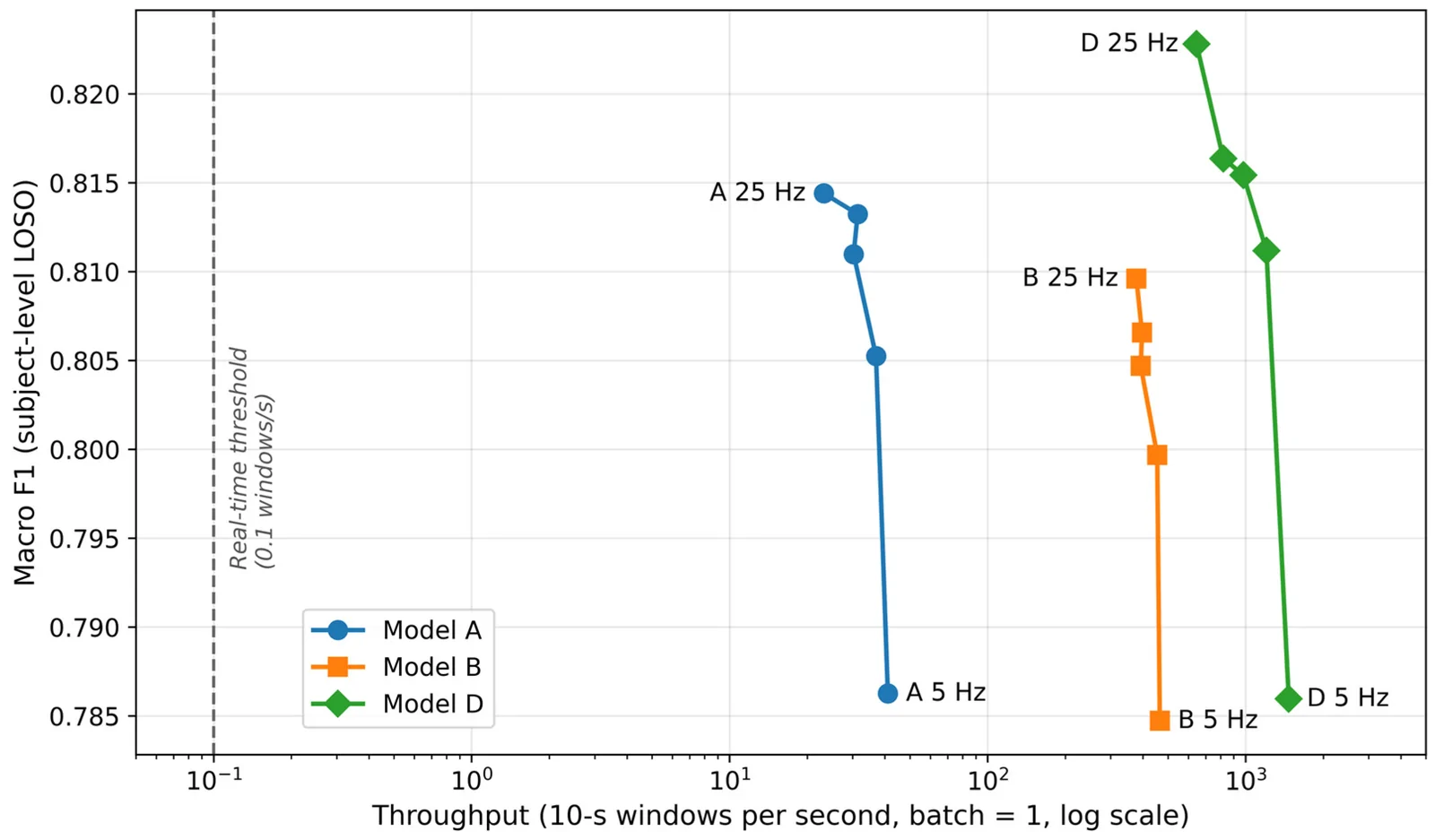
LOSO Macro F1 against throughput for Models A, B, and D across all sampling rates (batch = 1). Throughput is expressed in 10 s windows processed per second. The dashed vertical line marks the real-time threshold of 0.1 windows/s.

At 25 Hz on CPU, Model A processed 23 windows per second, Model B 377, and Model D 645 (Figure 9).

Under continuous monitoring, each animal generates 8640 windows per day. At the measured per-window latencies, processing one animal-day at 25 Hz corresponds to 373 s of single-thread CPU time for Model A, 23 s for Model B, and 13 s for Model D.

Expressed as transmitted payload per 10 s window, the reference configuration corresponds to 1000 bytes of handcrafted features in single precision, against 96 bytes for the 24-dimensional representation. The uncompressed raw six-channel signal at 25 Hz in 16-bit integer format corresponds to 3000 bytes.

Table 9 summarizes the combined reduction in transmitted feature dimensionality, input data volume, and inference latency relative to the Model A 25 Hz reference configuration.

## 4. Discussion

### 4.1. Compact Learned Representations Achieve Similar Performance with Fewer Features

Replacing the 250 handcrafted features of the baseline pipeline with the 24-dimensional representation of Model B reduced feature dimensionality by 90.4% and left classification performance essentially unchanged (Table 4). Under LOSO, the aggregate difference was 0.005, and the per-subject difference of 0.006 had a Tukey-adjusted 95% confidence interval of −0.027 to 0.040; both are small compared with the between-subject variability. The reduction was uniform across classes, at 0.004–0.006 in Rumination, Eating, and Other alike, so compression did not discard information specific to any one behavior.

More informative than the parity itself is that it was reached twice by extractors sharing no architectural components. Model B uses a multi-level attention mechanism. Model E combines three parallel CNN branches of differing kernel size with inter-channel correlation features and no attention at all. Parity achieved by a single architecture could reflect fortunate design, but parity achieved independently by two unrelated architectures suggests that 24 dimensions are sufficient to carry the information needed to separate these three behavioral states from a neck-mounted IMU. Such sufficiency is plausible given how handcrafted libraries are built. Generated combinatorially across channels, they are redundant by construction, and importance-based selection removes uninformative features without eliminating correlation among those retained [34,35].

The absolute performance obtained here is consistent with published values for accelerometer-based classification of feeding behaviors in small ruminants under window-level cross-validation. Mansbridge et al. [8] reported a mean F-score of 0.92 for a three-class collar-mounted model in sheep, and Hollevoet et al. [18] reported a balanced accuracy of 0.94 for seven activity classes, including grazing, in goats under stratified k-fold partitioning. For the three behavioral states used here, Pavlovic et al. [36] reported an F1 of 0.82 on held-out animals from neck-collar accelerometry in cattle. Direct numerical comparison across studies is limited by differences in species, sensor placement, and class definitions, and above all by validation design.

A second structural property was the ordering of class difficulty. Eating achieved the highest F1 and Other the lowest in every model under both validation schemes, and the between-model spread widened monotonically as class difficulty increased. Because this held across architectures differing substantially in overall performance, it reflects the structure of the problem and not any particular model. Eating in a pen-fed system involves large, sustained head and neck movements toward the feed trough, which produce a high-amplitude, readily separable signature at the collar. Rumination is a low-motion state in which the only consistent signal is the small rhythmic jaw movement of chewing [37]; against this quiet background, it shares much of its signal envelope with resting and other inactive states. Other is not a single behavior but a residual class aggregating several motor states, and its most frequent confusion was with Rumination (Figure 6), consistent with the two sharing a low-motion background. Architectural differences were therefore expressed almost entirely on the class whose boundary is least well defined.

### 4.2. Training Objective Has a Larger Effect than Extractor Choice

The ablation contrasts in Table 7 separate two design choices that are often discussed together. Substituting a learned extractor for the handcrafted feature battery, whether the attention network of Model B or the multi-scale CNN of Model E, changed Macro F1 by no more than 0.005 under either validation scheme. Substituting the training objective was far more consequential. The extractor architecture, the 24-dimensional bottleneck, and the downstream LightGBM classifier were held fixed while the objective moved from supervised classification to unsupervised reconstruction (Model E versus Model F). Macro F1 fell by 0.127 under LOSO and by 0.094 under 5-fold CV, at least an order of magnitude more. Within the space of designs examined here, representation quality was governed primarily by the objective the extractor was optimized against and not by the architecture producing it. Because the decoder is discarded at inference, the two pipelines are computationally equivalent, and they were trained under the same protocol apart from the loss.

The deficit was strongly class-dependent, smallest for Eating (0.040 under LOSO), intermediate for Rumination (0.123), and largest for Other (0.218), consistent with the class-difficulty ordering. Model F nonetheless remained well above the chance level of 0.333, so the reconstruction objective yielded an informative but less discriminative representation. One possible explanation not tested here is that a mean-squared reconstruction objective favors high-amplitude signal components, such as the head movements of eating, over the small-amplitude chewing pulses that separate rumination from other low-motion states [37]. Its performance should be read as a limitation of the specific reconstruction objective tested here, not as evidence against label-free representation learning in general [38,39,40].

### 4.3. Validation Design Reveals Substantial Between-Individual Variability

The two validation schemes produced systematically different estimates for every model, with aggregate Macro F1 decreasing by 0.044–0.095 from 5-fold CV to LOSO. This inflation under 5-fold CV arises from window-level partitioning: allocation is independent of subject identity and temporal position, so data from a single animal appear in both partitions. The annotation criterion compounds the effect: because only bouts of at least 180 s were retained, each contributes at least eighteen consecutive 10 s windows, and shuffling distributes windows from one bout across both partitions. A model evaluated this way can therefore exploit subject- and bout-specific characteristics that carry no information about the target behavior in deployment. Inflation of window-level estimates relative to animal-level validation has been reported repeatedly in cattle. Rahman et al. [16] found F-scores decreasing from 0.81–0.93 under stratified cross-validation to 0.15–0.80 under leave-one-animal-out validation on the same dataset, Riaboff et al. [7] likewise reported lower accuracy under cow-level than under sequence-level validation, and Wang et al. [15] reported Macro F1 decreasing from 0.89–0.94 under hold-out validation to 0.64–0.76 under leave-cow-out validation in beef cows fitted with neck-mounted accelerometers sampled at 25 Hz, as in the present study. The reduction of 0.044 to 0.095 observed here is smaller than that reported by Wang et al. [15], plausibly because the present dataset is class-balanced.

The dispersion statistics indicate the magnitude of this effect. Variation between folds was near zero, whereas variation between held-out subjects was one to two orders of magnitude larger. Fold-to-fold variation contains no between-animal information, because every fold draws windows from every subject; it therefore cannot inform generalization to new individuals. Model C provides a more direct probe. It differs from Model B only in replacing LightGBM with an instance-based KNN classifier, whose decision rule is the labels of the nearest training examples. Under window-level shuffling, those neighbors are likely to be windows adjacent in time and drawn from the same bout. Consistent with this, the penalty for substituting KNN was 0.011 under 5-fold CV but 0.030 under LOSO, and the amplification widened with class difficulty (0.008, 0.019, and 0.029 for Eating, Rumination, and Other). This contrast is not confounded by the labeler, whose KNN stage operated on handcrafted features rather than on the learned representation. Estimates obtained under window-level partitioning both here and elsewhere are therefore best read as measures of representational capacity and not of expected field performance [15].

Under LOSO, a second constraint emerged. Between-subject dispersion was large relative to the differences being compared, exceeding the full range of per-subject mean Macro F1 across Models A–E by roughly 5-fold. Because model comparisons are paired within subjects, that ratio describes the scale of individual variation; it does not establish that the models are indistinguishable. That conclusion rests on the mixed model, in which no pairwise contrast among Models A, B, D, and E reached significance and every confidence interval on their differences included zero. Across six repeated runs with different training seeds, aggregate Macro F1 varied with a standard deviation of 0.0005–0.0037, and Model B was below Model A in every run (mean difference 0.0054). Small differences of this kind are therefore reproducible for these 15 animals, but they are far smaller than the subject-level confidence intervals and do not indicate which model would perform better on new animals. The ranking by aggregate Macro F1 (with Model D coming first in all six runs) and by per-subject mean (with Model A coming first in five of six) differed, but by margins well within these confidence intervals.

Not all of the between-animal variation should be attributed to biological individuality. The models received the native IMU axes without per-subject orientation calibration, so differences in collar fit, resting orientation, and movement-induced rotation could have generated animal-specific offsets [19]. Each subject was also annotated by a single observer without overlap, so systematic differences in annotation practice were absorbed into the same term. Biological variation, sensor placement effects, and annotation effects are therefore intertwined here. Between-individual variation of this magnitude is consistent with earlier reports in goats [18] and cattle [15,16], and its dominance carries two consequences. First, accuracy is not a usable criterion for choosing among the leading models at this scale of evaluation, so selection must rest on operational properties. Second, further gains are more likely to come from per-individual adaptation—such as orientation normalization, rotation augmentation, or a short subject-specific calibration period after deployment—than from continued architectural search [41].

### 4.4. Computational Trade-Offs in Model Architecture and Sampling Rate

Because Models A, B, D, and E fall within 0.008 of one another in per-subject mean Macro F1, selection among them must rest on properties other than classification performance. Three such properties differ materially across the candidate architectures: the cost of updating a deployed model, the accessibility of the learned representation, and the complexity of the inference pipeline.

Update cost differs by construction between modular and end-to-end designs. In Models B and E, the feature extractor and the classifier are separate components, so extending the behavioral repertoire or adapting to a new site requires refitting only the downstream classifier while the extractor stays frozen. In Model D, any change to the label set requires retraining the entire network. This distinction matters most where inference runs on the collar itself [12,36]. The two designs therefore differ in what must be recomputed: gradient-based optimization of a whole network against a single tree-ensemble fit on precomputed features. On the same workstation, refitting the LightGBM classifier of Model B on precomputed 24-dimensional features took 4.8 s of CPU time (median of three runs), whereas end-to-end retraining of Model D took 368 s (36 epochs to early stopping). The comparison assumes that the frozen extractor remains adequate for the new label set, which was not tested. Where the behavioral repertoire is expected to expand, however, this structural difference is a more durable basis for selection than accuracy differences that do not reach significance. The remaining two criteria point in opposite directions. Models B and E expose a fixed 24-dimensional vector as an explicit interface, which could in principle be reused for additional classifiers or for transfer to related species or sensor placements, although no such transfer was evaluated here. Model D classifies in a single forward pass, with no external classifier and no version coordination between components, which has operational value where a fixed label set is deployed across many sites. These considerations identify which model suits which deployment pattern, not a single preferred model.

The latency figures reported here characterize relative computational cost on workstation hardware and do not by themselves establish feasibility on a collar-mounted microcontroller. A PyTorch model cannot execute directly on a Cortex-M-class device; the usual path is export to ONNX followed by conversion to an embedded runtime such as TensorFlow Lite Micro or CMSIS-NN, with 8-bit quantization. At that level of precision, the 21 K-parameter extractor of Model B would occupy roughly 21 KB and the 32 K-parameter network of Model D roughly 32 KB of parameter memory, which is within the flash budget of common Cortex-M4/M7 parts. The tree-ensemble stage of Models B and E, by contrast, comprises up to 1500 trees of 63 leaves and may occupy more memory than the extractor itself. If so, an end-to-end design such as Model D would be easier to fit on a microcontroller, whereas the modular designs may be better suited to gateway- or server-side inference, where the classifier can be refitted cheaply; neither the memory footprint of the tree ensemble nor the accuracy cost of quantization was measured here. Measured latency on the target device and the corresponding power draw remain to be established.

Sampling rate introduces a second, largely independent trade-off. Gains were strongly front-loaded. Under LOSO, operating at 15 Hz costs 0.003–0.007 in Macro F1 for a 40% reduction in sampled data volume, and 10 Hz costs 0.009–0.012 for a 60% reduction across Models A, B, D, and E. The diminishing gain above roughly 10–15 Hz is compatible with the slow rhythm of mastication, i.e., roughly one chewing stroke per second in ruminating cows [37] and somewhat faster in small ruminants [5], although the discriminative frequency components were not isolated here. This result therefore supports an empirical operating point, not a physiological threshold. Sampling rate and feature dimensionality act on different stages of the pipeline: the former reduces the raw data acquired and carried upstream, while the latter reduces the size of the representation passed onward and the cost of inference. The two are complementary, and their combined effect is summarized in Table 9.

### 4.5. Limitations and Future Work

Several limitations qualify these results. First, the behavioral labels were generated by a high-confidence semi-automatic procedure operating partly on the same IMU signals used for model development, so they were not fully independent of the model inputs. About 41% of the final windows were assigned by the automatic labeler, whose KNN stage uses the same handcrafted feature family as Model A; this potential circularity is therefore most relevant to Model A, and if anything biases the comparison against the learned representations that matched it. A class-stratified sample of 600 retained windows showed 99.3% agreement with synchronized video. That supports the reliability of the retained labels, but it does not eliminate the selection bias introduced by confidence filtering, since unanimity-based retention preferentially selects unambiguous windows. Inter-rater reliability of the manual annotation could not be assessed because each animal was annotated by a single observer, and intra-observer consistency over time was not tested.

Second, only Rumination and Eating episodes lasting at least 180 s were retained, and windows spanning transitions were excluded. Shorter episodes were not discarded but were treated as Other, so the residual class contains a small proportion of brief feeding and rumination bouts. The dataset therefore emphasizes sustained, clearly defined episodes and does not characterize performance on short, fragmented, or transitional events, which are likely the most difficult cases in continuous operation [3]. The Other class was drawn mainly from 7 fully annotated animal-days of 6 animals, so its diversity is narrower than that of the two focal classes, and 7 of the 15 LOSO subjects contributed no Other windows to their held-out partition. The dataset was also approximately balanced across classes. Under field conditions, the residual class occupies most of the day: housed goats spend roughly one sixth of the day eating [42] and about one fifth ruminating [43], so precision for the target behaviors would be lower in deployment. Reported metrics should be read as comparisons among models under matched conditions, not as estimates of field accuracy.

Third, generalizability beyond the present cohort remains uncertain. Data came from 19 Korean black goat wethers at a single site over one 9-day period. The LOSO analysis was restricted to the 15 animals meeting the minimum per-class requirement, so the subject-independent results do not characterize animals in which a target behavior is sparsely expressed. Robustness across breeds, demographic groups, housing systems, seasons, and collar-fitting conditions remains to be established.

Finally, the computational analyses were conducted offline on workstation hardware rather than on a production edge device, and two measurement asymmetries apply. The latency comparisons are implementation-specific, and Model A reused feature indices selected at 25 Hz at all lower rates with out-of-Nyquist features zero-filled, so its lower-rate performance is a conservative rather than a rate-optimized baseline. The bottleneck dimension was fixed at 24 in the main experiments, and the pilot comparison of 24, 48, and 96 dimensions (Section 2.4.3) was run under non-comparable conditions, so the results establish the sufficiency of 24 dimensions, not the intrinsic dimensionality of the task. The binary screening stage of the labeler was supplied by the sensor manufacturer, and its architecture is not disclosed, so the reliability of that stage is characterized empirically by agreement with synchronized video rather than by specification.

Within these limits, the study contributes three things that the existing literature on ruminant behavior classification rarely provides together: a controlled comparison in which feature source, classifier, and training objective are varied one at a time on identical data; window-level and animal-level validation reported side by side on the same dataset, quantifying the inflation that window-level partitioning produces; and the computational and communication costs (feature dimensionality, sampling rate, latency, and payload) that determine whether a model can run on a collar. For practitioners, the practical results are that a 24-dimensional learned representation is sufficient, that 10–15 Hz sampling retains most of the accuracy, and that among the leading models, the choice should be made based on update cost and inference pipeline rather than on accuracy differences that do not reach significance.

Three future research directions follow. First, fully independent, continuously human-annotated evaluation data are required to resolve label circularity and to evaluate performance under natural class prevalence. Second, the dominance of between-individual over between-model variation indicates that subject- or sensor-specific adaptation, together with on-device profiling and power measurement on target microcontroller hardware, is a higher-yield direction than further architectural refinement. Third, the deficit of Model F concerns reconstruction-based pretraining specifically; contrastive and masked-prediction objectives produce discriminative representations without reconstructing every input detail and may reduce annotation requirements in a way that a reconstruction objective does not [40,44,45].

## 5. Conclusions

This study compared six behavior classification models on neck-mounted six-axis IMU data from 19 Korean black goats, under both subject-level and window-level validation. Four conclusions follow. First, a 24-dimensional learned representation attained accuracy comparable to the 250-dimensional handcrafted feature set of the baseline pipeline, and two structurally unrelated extractors reached this parity. Replacing the supervised objective with unsupervised reconstruction, by contrast, reduced Macro F1 by 0.127 under LOSO and 0.094 under 5-fold CV, so within the designs examined, representation quality was governed primarily by the training objective and not by the extractor architecture. Second, window-level cross-validation produced systematically higher estimates than LOSO for every model. Window-level figures should therefore not be used for model selection or for claims about performance on new animals; they are reported here to quantify the inflation and to allow comparison with published results obtained under the same partitioning. Third, between-subject variability was roughly five times the range of per-subject mean performance across Models A–E, and no pairwise difference among the four leading models was statistically distinguishable from zero. Where accuracy cannot separate models, operational properties such as update cost, inference pipeline, and transmitted payload provide a more practical basis for selection; the latency figures reported here characterize relative computational cost on workstation hardware and await confirmation on target devices. Fourth, sampling-rate gains were front-loaded, with 10 to 15 Hz retaining most of the accuracy at 40 to 60% lower data volume. These operating points were established for sustained behavioral episodes in housed wethers of a single breed at one site, with labels from a semi-automatic procedure; their transferability to other breeds, housing systems, sensor placements, and natural class prevalence remains to be tested. Independent, continuously annotated evaluation data, evaluation under natural class prevalence, and per-individual adaptation are the immediate next steps.

## Figures and Tables

**Table 1:** Final dataset composition: retained per-class counts of 10 s windows after high-confidence filtering and class-balancing subsampling from 19 Korean black goats over nine recording days.

Class	Windows	Ratio (%)
Rumination	44,329	33.4
Eating	43,771	32.9
Other	44,787	33.7
Total	132,887	100.0

**Table 2:** Configuration of the six experimental models.

Model	Feature Extractor	Classifier	Features	Params	Model Size (KB)
A	Handcrafted	LightGBM	250	— ^a^	—
B	Multi-level Attention	LightGBM	24	~21 K	~84
C	Multi-level Attention	KNN (*k* = 5)	24	~21 K	~84
D	Temporal Attention + CNN	FC (end-to-end)	24 ^b^	~32 K	~128
E	Multi-scale CNN (Supervised)	LightGBM	24	~40 K	~160
F	Multi-scale CNN (Autoencoder)	LightGBM	24	~216 K ^c^	~864

Params refer to neural network parameters; model size is estimated as Params × 4 bytes (32-bit floating point) and excludes the LightGBM classifier. ^a^ Model A uses LightGBM alone, whose parameterization is not comparable to neural network parameter counts. ^b^ Dimensionality of the pooled summary vector; Model D classifies internally and exposes no feature vector. ^c^ Includes the decoder used during reconstruction training; the decoder is discarded at inference, so the deployed encoder matches Model E at approximately 40 K parameters.

**Table 3:** LightGBM classifier hyperparameters shared by Models A, B, E, and F.

Hyperparameter	Value	Notes
objective	multiclass	softmax over 3 classes
num_class	3	rumination/eating/other
metric	multi_logloss	training loss
boosting_type	gbdt	gradient boosting decision tree
num_leaves	63	tree complexity
learning_rate	0.05	shrinkage
feature_fraction	0.8	column subsampling per tree
bagging_fraction	0.8	row subsampling
bagging_freq	5	apply bagging every 5 iterations
num_boost_round	500	maximum boosting rounds
early_stopping	50 rounds	patience on training loss
seed	42	reproducibility (NumPy/PyTorch/LightGBM)
n_jobs	−1	all available CPU cores

**Table 4:** Aggregate and per-partition Macro F1 of all six models under both validation schemes.

Model	LOSO Macro F1 (Aggregate)	LOSO Macro F1 (Six Runs, Mean ± SD)	LOSO Per-Subject Mean ± SD (*n* = 15)	Five-Fold Macro F1 (Aggregate)	Five-Fold Mean ± SD (*n* = 5)
A	0.8144	0.8141 ± 0.0005	0.8132 ± 0.1339	0.8758	0.8758 ± 0.0018
B	0.8096	0.8087 ± 0.0022	0.8071 ± 0.1342	0.8757	0.8757 ± 0.0021
C	0.7800	0.7816 ± 0.0023	0.7880 ± 0.1496	0.8649	0.8649 ± 0.0020
D	0.8228	0.8203 ± 0.0037	0.8071 ± 0.1181	0.8668	0.8668 ± 0.0011
E	0.8124	0.8101 ± 0.0009	0.8048 ± 0.1310	0.8748	0.8748 ± 0.0008
F	0.6855	0.6792 ± 0.0021	0.7356 ± 0.1739	0.7806	0.7805 ± 0.0079

Per-subject Macro F1 is the mean of per-class F1 over the classes present in each subject’s held-out data; 7 of the 15 subjects contributed no Other windows. Six-run values are from repeated LOSO runs with training seeds 42–47 (Table S5); all other values are from the original run.

**Table 5:** Per-class F1 under subject-level LOSO.

Model	Features	Macro F1	Rumination F1	Eating F1	Other F1
A	250	0.814	0.824	0.883	0.736
B	24	0.810	0.819	0.879	0.730
C	24	0.780	0.793	0.863	0.684
D	24	0.823	0.828	0.884	0.756
E	24	0.812	0.814	0.886	0.737
F	24	0.685	0.691	0.846	0.519

**Table 6:** Per-class F1 under stratified 5-fold cross-validation.

Model	Features	Macro F1	Rumination F1	Eating F1	Other F1
A	250	0.876	0.886	0.917	0.824
B	24	0.876	0.886	0.917	0.824
C	24	0.865	0.879	0.909	0.807
D	24	0.867	0.872	0.913	0.816
E	24	0.875	0.877	0.924	0.824
F	24	0.781	0.792	0.874	0.676

**Table 7:** Change in aggregate Macro F1 between model pairs differing primarily in a single design variable.

Contrast	Variable Changed	Δ LOSO	Δ 5-Fold
A → B	Extractor: handcrafted-250 → attention-24	−0.0048	−0.0001
A → E	Extractor: handcrafted-250 → CNN-24	−0.0020	−0.0010
E → F	Objective: supervised → reconstruction	−0.1269	−0.0942
B → C	Classifier: LightGBM → KNN	−0.0296	−0.0108

Training data and evaluation protocol are identical within each contrast.

**Table 8:** Per-sample inference latency (batch = 1, median of 50 timed runs after 10 warm-up runs).

Hz	A CPU (ms)	B CPU (ms)	B GPU (ms)	D CPU (ms)	D GPU (ms)
5	24.35	2.15	3.81	0.68	1.08
10	27.06	2.20	4.01	0.83	0.97
15	33.05	2.55	3.89	1.02	0.98
20	31.88	2.52	3.99	1.22	0.97
25	43.16	2.65	4.10	1.55	1.01

CPU measurements use a single thread. Model A was measured on CPU only.

**Table 9:** Aggregate reduction in feature dimensionality, input data volume, and inference latency.

Config	Features	LOSO Macro F1	Δ	Feature red.	Input Data red.	Speedup vs. A@25 Hz	Speedup vs. A, Same Rate
A 25 Hz (ref)	250	0.814	—	—	—	1.0×	—
B 25 Hz	24	0.810	−0.005	90.4%	0%	16.3×	16.3×
B 20 Hz	24	0.807	−0.008	90.4%	20%	17.1×	12.7×
B 15 Hz	24	0.805	−0.010	90.4%	40%	16.9×	13.0×
B 10 Hz	24	0.800	−0.015	90.4%	60%	19.6×	12.3×
B 5 Hz	24	0.785	−0.030	90.4%	80%	20.1×	11.3×
D 25 Hz	24	0.823	+0.008	90.4%	0%	27.8×	27.8×
D 20 Hz	24	0.816	+0.002	90.4%	20%	35.2×	26.0×
D 15 Hz	24	0.815	+0.001	90.4%	40%	42.3×	32.4×
D 10 Hz	24	0.811	−0.003	90.4%	60%	51.9×	32.6×
D 5 Hz	24	0.786	−0.028	90.4%	80%	63.9×	36.1×

Δ is the change in LOSO Macro F1 relative to the Model A 25 Hz reference. Δ and speedup ratios were computed from unrounded values.

## Data Availability

The original contributions of this study are presented within the article. For further information or requests regarding the data, please contact the corresponding author.

## Supplementary Materials

The following supporting information can be downloaded at https://www.mdpi.com/article/10.3390/s26206424/s1, Table S1: Aggregate Macro F1 for all six models at each sampling rate under both validation schemes; Table S2: Macro and per-class F1 for all six models at each sampling rate under both validation schemes; Table S3: Per-class precision and recall for all six models under both validation schemes at 25 Hz; Table S4: Number of retained 10 s windows per subject and class in the final dataset; Table S5: Aggregate LOSO Macro F1 and per-class F1 of the six models in six repeated runs with different training seeds.

## Abbreviations

The following abbreviations are used in this manuscript:

BN	Batch normalization
CI	Confidence interval
CNN	Convolutional neural network
CPU	Central processing unit
CUDA	Compute Unified Device Architecture
CV	Cross-validation
FC	Fully connected
GAP	Global average pooling
GELU	Gaussian error linear unit
GPU	Graphics processing unit
I/O	Input/output
IMU	Inertial measurement unit
KNN	*k*-nearest neighbors
LightGBM	Light Gradient Boosting Machine
LOSO	Leave-one-subject-out
LTS	Long-term support
MHA	Multi-head self-attention
MSE	Mean squared error
ONNX	Open Neural Network Exchange
RAM	Random-access memory
RDA	Rural Development Administration
ReLU	Rectified linear unit
SD	Standard deviation
