# Supplementary material for: Handcrafted Features Versus Learned Representations for IMU-Based Goat Behavior Classification: Accuracy, Validation Design, and Computational Cost
Source: Sensors (Basel). 2026 Oct 11;26(20):6424. doi: 10.3390/s26206424 (PMC13708839; doi:10.3390/s26206424)
Supplement: Supplementary file 1 [file sensors-26-06424-s001.zip › sensors-4553923-supplementary.pdf]

Supplementary Materials

Table S1. Aggregate Macro F1 for all six models at each sampling rate under both validation schemes.

Sampling rate (Hz)	Model	LOSO Macro F1	5-fold CV Macro F1	Gap (5-fold – LOSO)
5	A	0.786	0.854	0.067
5	B	0.785	0.845	0.060
5	C	0.748	0.822	0.074
5	D	0.786	0.842	0.056
5	E	0.780	0.845	0.065
5	F	0.678	0.781	0.104
10	A	0.805	0.867	0.062
10	B	0.800	0.862	0.063
10	C	0.774	0.848	0.074
10	D	0.811	0.861	0.050
10	E	0.803	0.865	0.062
10	F	0.687	0.780	0.093
15	A	0.811	0.873	0.062
15	B	0.805	0.871	0.066
15	C	0.778	0.859	0.081
15	D	0.815	0.865	0.050
15	E	0.805	0.871	0.066
15	F	0.687	0.793	0.106
20	A	0.813	0.875	0.062
20	B	0.807	0.873	0.066
20	C	0.778	0.862	0.084
20	D	0.816	0.868	0.051
20	E	0.809	0.874	0.065
20	F	0.677	0.792	0.115
25	A	0.814	0.876	0.061
25	B	0.810	0.876	0.066
25	C	0.780	0.865	0.085
25	D	0.823	0.867	0.044
25	E	0.812	0.875	0.062
25	F	0.685	0.781	0.095

LOSO, leave-one-subject-out over the 15 subjects meeting the minimum per-class requirement; 5-fold CV, stratified 5-fold cross-validation. Aggregate Macro F1 was computed on predictions pooled across all held-out partitions. Gaps were computed from unrounded values and can therefore differ by 0.001 from the difference of the rounded entries. Values at 25 Hz correspond to Table 4.

Table S2. Macro and per-class F1 for all six models at each sampling rate under both validation schemes.

Model	Hz	LOSO Macro	LOSO Rum	LOSO Eat	LOSO Oth	5-fold Macro	5-fold Rum	5-fold Eat	5-fold Oth
A	5	0.786	0.794	0.873	0.692	0.854	0.862	0.908	0.791
A	10	0.805	0.818	0.877	0.721	0.867	0.876	0.914	0.810
A	15	0.811	0.822	0.880	0.731	0.873	0.883	0.915	0.820
A	20	0.813	0.824	0.881	0.735	0.875	0.885	0.917	0.823
A	25	0.814	0.824	0.883	0.736	0.876	0.886	0.917	0.824
B	5	0.785	0.797	0.865	0.692	0.845	0.858	0.901	0.777
B	10	0.800	0.811	0.875	0.713	0.862	0.874	0.910	0.803
B	15	0.805	0.818	0.874	0.722	0.871	0.883	0.913	0.815
B	20	0.807	0.819	0.876	0.724	0.873	0.884	0.915	0.820
B	25	0.810	0.819	0.879	0.730	0.876	0.886	0.917	0.824
C	5	0.748	0.764	0.847	0.633	0.822	0.841	0.884	0.741
C	10	0.774	0.790	0.858	0.674	0.848	0.862	0.901	0.782
C	15	0.778	0.793	0.861	0.681	0.859	0.873	0.906	0.798
C	20	0.778	0.796	0.858	0.680	0.862	0.876	0.907	0.802
C	25	0.780	0.793	0.863	0.684	0.865	0.879	0.909	0.807
D	5	0.786	0.788	0.875	0.695	0.842	0.846	0.904	0.777
D	10	0.811	0.817	0.881	0.736	0.861	0.866	0.914	0.804
D	15	0.815	0.821	0.883	0.742	0.865	0.868	0.915	0.813
D	20	0.816	0.818	0.888	0.743	0.868	0.872	0.915	0.815
D	25	0.823	0.828	0.884	0.756	0.867	0.872	0.913	0.816
E	5	0.780	0.779	0.873	0.687	0.845	0.845	0.911	0.779
E	10	0.803	0.803	0.882	0.722	0.865	0.867	0.919	0.808
E	15	0.805	0.808	0.885	0.723	0.871	0.874	0.922	0.818
E	20	0.809	0.810	0.886	0.732	0.874	0.877	0.923	0.822
E	25	0.812	0.814	0.886	0.737	0.875	0.877	0.924	0.824
F	5	0.678	0.684	0.842	0.507	0.781	0.791	0.876	0.676
F	10	0.687	0.683	0.842	0.535	0.780	0.791	0.875	0.675
F	15	0.687	0.677	0.841	0.544	0.793	0.802	0.878	0.700
F	20	0.677	0.675	0.837	0.518	0.792	0.800	0.878	0.697
F	25	0.685	0.691	0.846	0.519	0.781	0.792	0.874	0.676

Rum, Rumination; Eat, Eating; Oth, Other. Values at 25 Hz correspond to Tables 5 and 6.

Table S3. Per-class precision and recall for all six models under both validation schemes at 25 Hz.

Scheme	Model	Rum P	Rum R	Eat P	Eat R	Oth P	Oth R
LOSO	A	0.805	0.845	0.886	0.878	0.749	0.723
LOSO	B	0.794	0.843	0.884	0.871	0.740	0.709
LOSO	C	0.763	0.825	0.862	0.865	0.711	0.656
LOSO	D	0.824	0.830	0.888	0.872	0.748	0.757
LOSO	E	0.790	0.841	0.888	0.881	0.754	0.716
LOSO	F	0.636	0.716	0.831	0.846	0.563	0.489
5-fold CV	A	0.891	0.881	0.919	0.916	0.819	0.830
5-fold CV	B	0.889	0.885	0.920	0.914	0.820	0.828
5-fold CV	C	0.873	0.885	0.907	0.912	0.816	0.799
5-fold CV	D	0.891	0.854	0.920	0.907	0.794	0.839
5-fold CV	E	0.879	0.875	0.925	0.922	0.820	0.827
5-fold CV	F	0.775	0.812	0.873	0.878	0.701	0.664

Rum, Rumination; Eat, Eating; Oth, Other; P, precision; R, recall. Values are from an independent re-execution of both protocols under identical seeds (the same run as Figure 6). Because GPU execution was not deterministic, F1 computed from these values differs from Tables 5 and 6 by at most 0.006 for Models A–E and by at most 0.018 for Model F.

Table S4. Number of retained 10-s windows per subject and class in the final dataset.

Subject	Days	Rumination	Eating	Other	Total	Used in LOSO
Subject 01	8	3,093	1,017	0	4,110	Yes
Subject 02	6	642	950	0	1,592	Yes
Subject 03	9	4,638	10,907	1,001	16,546	Yes
Subject 04	9	4,825	10,625	1,134	16,584	Yes
Subject 05	6	3,769	4,750	5,643	14,162	Yes
Subject 06	8	4,825	6,196	5,322	16,343	Yes
Subject 07	8	719	531	0	1,250	Yes
Subject 08	8	1,743	661	0	2,404	Yes
Subject 09	4	1,182	151	7,476	8,809	Yes
Subject 10	7	2,362	969	0	3,331	Yes
Subject 11	8	1,358	1,014	0	2,372	Yes
Subject 12	8	1,427	1,097	0	2,524	Yes
Subject 13	4	2,550	1,466	12,156	16,172	Yes
Subject 14	9	4,198	2,311	4,658	11,167	Yes
Subject 15	8	2,972	1,126	7,397	11,495	Yes
Subject 16	4	349	0	0	349	No
Subject 17	6	214	0	0	214	No
Subject 18	7	268	0	0	268	No
Subject 19	8	3,195	0	0	3,195	No
Total	135	44,329	43,771	44,787	132,887	

Days, number of recording days contributing windows. Subjects 01–15 were used in LOSO; Subjects 16–19 did not meet the minimum of 50 windows in both focal classes. Subject numbers are anonymized identifiers.

Table S5. Aggregate LOSO Macro F1 and per-class F1 of the six models in six repeated runs with different training seeds.

	Model A	Model B	Model C	Model D	Model E	Model F
Aggregate Macro F1						
Seed 42*	0.8141	0.8064	0.7794	0.8200	0.8111	0.6783
Seed 43	0.8140	0.8088	0.7841	0.8187	0.8113	0.6789
Seed 44	0.8147	0.8113	0.7832	0.8214	0.8097	0.6768
Seed 45	0.8140	0.8088	0.7833	0.8249	0.8098	0.6776
Seed 46	0.8146	0.8060	0.7783	0.8227	0.8096	0.6815
Seed 47	0.8134	0.8109	0.7812	0.8141	0.8093	0.6819
Mean	0.8141	0.8087	0.7816	0.8203	0.8101	0.6792
SD	0.0005	0.0022	0.0023	0.0037	0.0009	0.0021
95% CI	0.8136–0.8146	0.8064–0.8110	0.7791–0.7840	0.8164–0.8242	0.8092–0.8110	0.6770–0.6814
Per-subject mean Macro F1						
Mean	0.8127	0.8065	0.7874	0.8066	0.8041	0.7299
SD	0.0004	0.0013	0.0012	0.0042	0.0011	0.0060
Per-class F1, mean						
Rumination	0.8242	0.8192	0.7931	0.8234	0.8126	0.6775
Eating	0.8825	0.8784	0.8645	0.8852	0.8860	0.8393
Other	0.7358	0.7285	0.6871	0.7524	0.7318	0.5206

Each run repeated the full LOSO protocol at 25 Hz over the 15 held-out subjects with the training seed shown; the dataset and the subject partitions were unchanged. * The run with seed 42 is the re-execution used for Figure 6 and Table S3; the values in Tables 4–7 come from the original execution with the same seed, which differs from it because GPU kernel execution was not deterministic (Section 2.10). Mean, SD and the 95% confidence interval of the mean (t distribution, n = 6) are computed across runs; they describe run-to-run variation for these 15 subjects, not uncertainty for new animals. The SD of per-class F1 across runs was at most 0.0060 for every class and model. Per-subject Macro F1 is averaged over the classes present in each subject's held-out data.
